# Modelling seasonal dynamics, population stability, and pest control in *Aedes japonicus japonicus* (Diptera: Culicidae)

**DOI:** 10.1186/s13071-019-3366-2

**Published:** 2019-03-25

**Authors:** Andreas Wieser, Friederike Reuss, Aidin Niamir, Ruth Müller, Robert B. O’Hara, Markus Pfenninger

**Affiliations:** 1 Senckenberg Biodiversity and Climate Research Centre, Senckenberganlage 25, 60325 Frankfurt am Main, Germany; 2 Institute of Organismic and Molecular Evolution (iOME), Johannes Gutenberg University, Gresemundweg 2, 55128 Mainz, Germany; 3 Centre for Biodiversity Dynamics, and Department of Mathematical Sciences, Norwegian University of Science and Technology NTNU, Sentralbygg 2, Gløshaugen, 7491 Trondheim, Norway; 4 Institute for Ecology, Evolution and Diversity, Faculty of Biological Sciences, Goethe University, Max-von-Laue-Straße 9, 60438 Frankfurt am Main, Germany; 5 Faculty of Medicine, Institute of Occupational Medicine, Social Medicine and Environmental Medicine, Goethe University, Theodor-Stern-Kai 7, 60590 Frankfurt am Main, Germany; 6 Unit of Entomology, Institute of Tropical Medicine, Nationalenstraat 155, 2000 Antwerp, Belgium

**Keywords:** StagePop, Population continuity, Differential delay equation, Stage-structured model, Asian bush mosquito, Invasive species

## Abstract

**Background:**

The invasive temperate mosquito *Aedes japonicus japonicus* is a potential vector for various infectious diseases and therefore a target of vector control measures. Even though established in Germany, it is unclear whether the species has already reached its full distribution potential. The possible range of the species, its annual population dynamics, the success of vector control measures and future expansions due to climate change still remain poorly understood. While numerous studies on occurrence have been conducted, they used mainly presence data from relatively few locations. In contrast, we used experimental life history data to model the dynamics of a continuous stage-structured population to infer potential seasonal densities and ask whether stable populations are likely to establish over a period of more than one year. In addition, we used climate change models to infer future ranges. Finally, we evaluated the effectiveness of various stage-specific vector control measures.

**Results:**

*Aedes j. japonicus* has already established stable populations in the southwest and west of Germany. Our models predict a spread of *Ae. j. japonicus* beyond the currently observed range, but likely not much further eastwards under current climatic conditions. Climate change models, however, will expand this range substantially and higher annual densities can be expected. Applying vector control measures to oviposition, survival of eggs, larvae or adults showed that application of adulticides for 30 days between late spring and early autumn, while ambient temperatures are above 9 °C, can reduce population density by 75%. Continuous application of larvicide showed similar results in population reduction. Most importantly, we showed that with the consequent application of a mixed strategy, it should be possible to significantly reduce or even extinguish existing populations with reasonable effort.

**Conclusion:**

Our study provides valuable insights into the mechanisms concerning the establishment of stable populations in invasive species. In order to minimise the hazard to public health, we recommend vector control measures to be applied in ‘high risk areas’ which are predicted to allow establishment of stable populations to establish.

**Electronic supplementary material:**

The online version of this article (10.1186/s13071-019-3366-2) contains supplementary material, which is available to authorized users.

## Background

Accidentally displacing and intentionally introducing individuals of a species through anthropogenic activities, such as trade, migration or traffic, has led to numerous incidences of non-native species colonising new environments outside their previous ranges. While there are several definitions of invasive species [1], Williamson and Fitter’s [2] “tens rule” separates these alien species in the categories (i) imported, (ii) introduced, (iii) established, and (iv) pest, with a transition probability between each category of approximately 10%. Alien species that become pests cause damage to the receiving ecosystem, or health hazards to human populations [3]. Invasive mosquito species in Europe have been identified as a significant risk to public health [3], sometimes acting as vectors for exotic or (re-)emerging disease agents such as La Crosse virus, dengue virus, West Nile virus or Zika virus [4–7].

The Asian bush mosquito *Aedes japonicus japonicus* (Diptera: Culicidae) native to temperate East Asia [8–10] was initially documented in Europe in 2000 (France [11]) and 2002 (Belgium [12]). It is listed as an invasive species on the Global Invasive Species Database [13] and occurrences in Europe were reported in the Netherlands, Belgium, France, Germany, Switzerland, Liechtenstein, Austria, Italy, Hungary, Slovenia, Croatia [14], and most recently Spain [15]. Genetic analyses support multiple introductions as well as expansion of the existing populations [16, 17]. It has become a well-established alien species in Germany [18, 19] (probably because of its origin from temperate regions and an accompanying pre-adaptation to temperature fluctuations and pronounced seasonality, see Additional file 1: Figure S9). Recent reports suggest spread or an independent introduction into Austria [20].

Some studies argue that invasive mosquito species must exhibit an advantage in larval competition, further aiding their successful establishment [21]. However, this issue is still heavily debated as examples of the opposite, i.e. advantage in larval competition for endemic species, can be observed [22]. Studies conducted in the USA suggested the invasion of *Ae. j. japonicus* coincided with a reduction of native container-inhabiting mosquitoes [23], hinting at interspecific larval competition. However, laboratory experiments could not corroborate these results [24].

As verified in the laboratory, *Ae. j. japonicus* is a potential vector of Japanese encephalitis virus [25], West Nile virus [26], chikungunya virus and dengue virus [27]. Thus, further population expansion could pose a risk to public health in the future. This makes the mosquito species a target of vector control measures to limit growth and further spread of the introduced populations [28].

Pest control measures usually target certain stages in the life-cycle of mosquitoes [29], e.g. the larvicide *Bacillus thuringiensis israelensis* (Bti) affects larval stages [30]. There are several other types of control tools, targeting either oviposition or adults [31–33]. Alternatively, sterile insect techniques (SIT) using genetically modified mosquitoes enable the disruption of reproduction rates [34]. The effect of these vector control measures on natural population dynamics is often hard to discern, but data-driven modelling of population dynamics might help to evaluate the potential success of specific control strategies [35]. In order to examine effects of specific control measures targeting different life stages we categorise these strategies into (i) ovicides targeting the egg stage (e.g. RNAi-methods, physical removal of containers after oviposition); (ii) larvicides targeting the larval stage (e.g. Bti; cyclopoid copepods); (iii) adulticides targeting adults (e.g. space spraying with chemical insecticides); and (iv) deterrents to oviposition (physical removal of containers before oviposition; treatment of containers with essential oils having repellent effects; SIT) targeting reproduction rates (e.g. male treatment by radiation, chemicals or genetic modifications) [30].

As climate is shifting, its impact on local ecosystems becomes an important issue not only for endemic species. Its effect on invasive species is of special interest, as their response may differ from native species [36, 37]. If climate change favours an alien species, particularly a potential disease vector, the implications for public health may become gravely important.

Stage-structured population models are a useful tool to predict population dynamics, especially in multivoltine insects and other invertebrates with distinct metamorphosis events [38]. Their deterministic nature offers an elegant solution to model natural life cycles of big populations in which random environmental fluctuations can be ignored. In many temperate insect species, generations at the beginning of the year are synchronised due to diapause during winter. However, because of individual variation in development, initially discrete generations become increasingly ‘smeared’ over the course of the year. Accordingly, we observe a change from discrete to overlapping generations [39]. This effect can be modelled through delay-differential equations [40] in which the rate of change of the state variable at any given time depends on its value at an earlier time.

At the example of *Ae. j. japonicus* in Germany, we aim to deepen the understanding of (i) how this non-native species can become permanently established in a new environment; (ii) how best to combat established populations; and (iii) what impact climate change will have on the potential distribution of the species. Therefore, we study the transition from ‘introduced’ to ‘established’ status of invasive species [2, 41, 42] by evaluating the potential to establish stable populations in a new environment. We assume the potential for stable population continuity, i.e. persistence of a population over the course of more than one year, is a central factor in the transition from an introduced to an established alien species. In the temperate climate of Germany this pertains to the potential of a population to survive and thrive after a prolonged cold period.

Our goal is to identify ‘high risk areas’ in Germany, where *Ae. j. japonicus* either is already established or could potentially establish long-term stable populations. Furthermore, we predict the success of different stage-specific control measures to limit or even eradicate these populations.

## Methods

### Model description

In our model the life-cycle of *Ae. j. japonicus* is defined by five life stages: eggs, larvae, pupae, sexually immature adults before the intake of a blood meal (hereafter called ‘premature’), and reproducing adults. The length of stages, given by the development rates, as well as mortality rates vary not only between stages but also heavily depend on ambient temperature, e.g. larval development is faster at higher temperatures. The required data on life-history traits was obtained from life-cycle experiments published in Reuss et al. [43], as well as the experiments described below. We fitted functions for parameters of the following life-history traits: temperature dependent development, mortality, reproduction rates, as well as density-dependent larval mortality (see Additional file 1: Table S2 and Additional file 1: Text S2.2–2.6 for detailed description). For temperatures below 7 °C we deviated from these functions, still in concordance with results from [43], by prolonging egg development to account for diapause.

We used the R-package *stagePop* [44] to model annual population dynamics of stage-structured populations. The package is based on model formulations by Nisbet & Gurney [45], using delay-differential equations for a continuous time population dynamics model. The model is based on the following equation [46], where the change of the state variable, i.e. the number of individuals *N*_*i*_ in stage *i* over time *t* is described as

1 $$\frac{{dN_{i} \left( t \right)}}{dt} = R_{i} \left( t \right) - M_{i} \left( t \right) - \delta_{i} \left( t \right)N_{i} \left( t \right),$$

where *R*_*i*_(t) corresponds to the recruitment into stage *i*, *M*_*i*_(t) describes maturation from stage *i*, and *δ*_*i*_(t) is the per capita loss rate of individuals in stage *i*, in our case the through-stage mortality rate. Note that per capita loss rate of individuals in the egg stage includes any factor that inhibits progression to the next stage, not exclusively mortality of pharate larvae. For simplicity, it is still referred to as ‘egg mortality’. The rate of maturation, *M*_*i*_(t) is given by

2 $$M_{i} \left( t \right) = R_{i} \left( {t - \tau_{i} \left( t \right)} \right)P_{i} \left( t \right)\left( {1 - \frac{{d\tau_{i} \left( t \right)}}{dt}} \right),$$

where *R*_*i*_(t) corresponds to the recruitment into stage *i*, *P*_*i*_(t) denotes the fraction of individuals entering stage *i* at *t *− *τ*_*i*_(t) that has survived to time *t*. Maturation from one stage corresponds to recruitment into the next stage. Recruitment into the first, i.e. egg stage is defined by the birth rate. Note that in our model, we disregard immigration and emigration terms. For a more detailed description, we refer to the *stagePop* manual and Nisbet & Gurney’s work [40, 44–46]. The R-code of the model is provided in Additional file 1: Text S1.1–1.3.

**Fig. 1:**
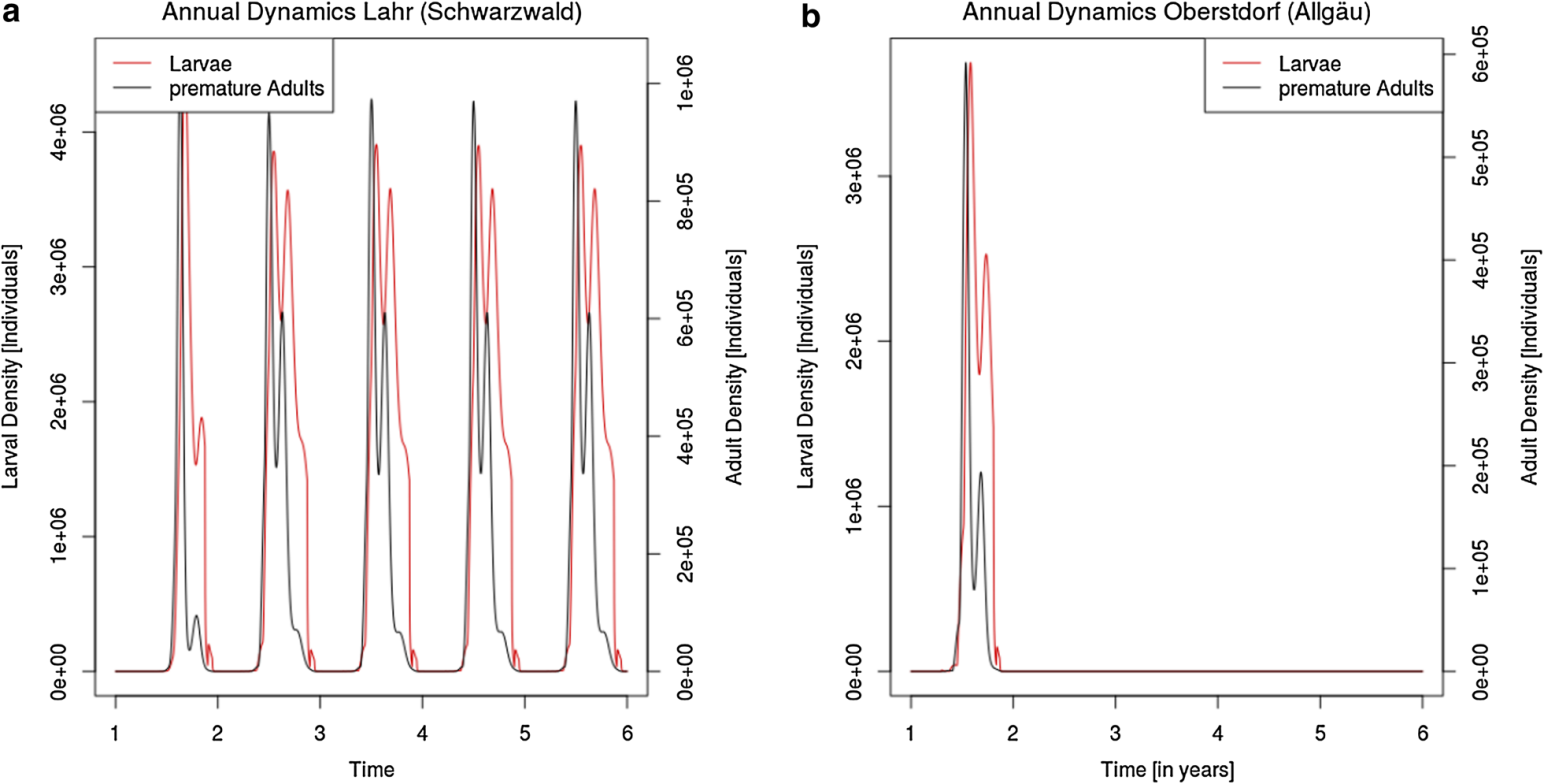
Annual population dynamics of two life stages (larvae and adults) of *Aedes j. japonicus* at two locations in Germany. **a** In Lahr, population can persist over multiple years. **b** In Oberstdorf, population becomes extinct. Local temperature is based on data provided by Deutscher Wetterdienst (DWD) [78]

The deterministic population dynamics model was performed independently for every cell of a gridded map of Germany using temperature as variable input (details on grids can be found in the Scenario section below). All other parameters were kept constant over all iterations of the model. In order to allow population dynamics to reach a stable equilibrium, we ran the simulations for 5 years, reusing the same temperature function. These repetitions minimise the differences between years as stable dynamics establish after the third year (Fig. 1). We calculated the cumulative population density of larvae in the fifth year and normalised the values by the highest observed population density.

### Parameter elicitation

#### Life history traits

We used published life-cycle data of *Ae. j. japonicus* [43] to obtain the necessary parameters for our model, as well as added newly gathered data on temperature-dependent egg hatching and intraspecific larval density effects.

#### Egg development and loss rate

To obtain data on the earliest stage, eggs of *Ae. j. japonicus* were collected from June 3–16, 2017 in Biberach (Baden, Germany), by means of pressboard sticks serving as oviposition substrate. Eggs were stored in sealed plastic bags at 25 °C until June 30, 2017. Circles with 2 cm diameter were cut out from coffee filters and placed on the bottom of 80 100 ml plastic cups and soaked with 200 μl deionised water. Twenty eggs were placed on each filter paper (in total 1600 eggs). After flooding with 80 ml deionised water, 20 cups each were placed in one of the four tested temperatures (0, 10, 20 and 30 °C) which cover the complete temperature range in which the life-cycle of *Ae. j. japonicus* can be completed [43]. Daily, the occurrence of hatched larvae was observed, and larvae removed. The percentage of eggs that did not hatch was used to calculate the per capita loss rate of individuals during the egg stage.

We used recently published wing length measurements [43] of adult females reared at 14 temperatures (between 10–31 °C) and utilised the correlation between wing length and fecundity as stated in [47] to obtain a temperature-dependent birth rate.

#### Larval density effect

The effect of intraspecific larval crowding on mortality, i.e. the density effect, was assessed in a laboratory experiment. We placed 10, 20, 40, 80 and 160 larvae of *Ae. j. japonicus* younger than 24 h in 1-litre plastic cups with deionised water as larval medium and fed 10 mg TetraMin (Tetra, Melle, Germany) per capita, according to [48]. The number of emerged adults was evaluated on a daily basis and larval mortality was calculated.

#### Temperature data and raster calculation

As *Ae. j. japonicus* spends its first three stages (egg, larva, pupa) mainly in containers with water (e.g. vases, buckets, rain barrels), experienced temperature can vary depending on container size, location, material, and even colour [22]. For the large scale of our study, ambient air temperature was the only available proxy. We used three datasets (CHELSA [49, 50], Worldclim [51] and E-OBS [52]) to accommodate different parts of our study, depending on the scenario (see sections below).

We fitted a sinusoidal function to approximate the temperature data for every cell on the raster (see Additional file 1: Text S2.1). This transformation of temperature data to a temperature function smooths over extreme events, such as short-term freezing or summer heat-waves (see Additional file 1: Figure S1). However, as Reuss et al. [43] state, larvae will not survive a period of three consecutive days below 0 °C, which is reflected in our model by raising larval mortality when temperature is below 0 °C. In warmer regions, e.g. along the Rhine, our fitted function will prevent temperatures to drop below 0 °C (lowest point in Lahr (Baden) is approximately 2.13 °C, Additional file 1: Figure S1). Nonetheless, at least one freezing event can be expected for every grid cell throughout Germany. Thus, larval mortalities were raised in all grid cells at the 3 days of lowest temperature, regardless of the values from the approximated temperature curve (for a more detailed discussion of the winter mortality conditions see Additional file 1: Text S5).

### Scenarios

#### Current condition

To obtain a detailed distribution of areas offering the conditions for stable population establishment of *Ae. j. japonicus* under current climatic conditions, we chose temperature data from the CHELSA dataset [49, 50], offering a resolution of 30′′, or approximately 1 km. We fitted the temperature function to average monthly temperatures over a period of 20 years (1993–2013). Simulations were conducted according to the description in the model section. As the CHELSA dataset is of a very high resolution (> 10^6^ cells), computation time for the current condition was several weeks. Thus, we chose lower resolutions for our climate change (2.5′, 46,000 cells) and vector control (0.25°, 10^3^ cells) scenarios as they required multiple runs of the simulations (overall 51 runs across all scenarios). For the following scenarios on climate change and vector control, we chose the highest feasible spatial resolutions.

#### Climate change

For the future model, we calculated mean monthly temperatures of the Coupled Model Intercomparison Project (CMIP) 2.5′ models (approximately 4.5 km at equator), specifically we used Community Climate System Model (CCSM4) representative concentration pathway (RCP) mitigation scenarios RCP4.5 and RCP8.5 for the time period 2041–2060 (Worldclim database; http://worldclim.org/CMIP5v1, [51, 53]). These scenarios represent two possible changes in radiative forcing values (in W/m^2^) projected for the year 2100 relative to pre-industrial conditions [54]. Radiative forcing refers to imbalance between incoming and outgoing radiation to the atmosphere caused by changes in atmospheric constituents (among CO_2_). All other conditions of the stage-structured population model were chosen according to the current condition scenario.

### Vector control measures

We chose a quasi-equal area resolution of 25 km of daily observed data (1997–2017) provided by the ENSEMBLES project to model success of vector control measures (E-OBS version 14.0; http://ensembles-eu.metoffice.com, [52]). While this dataset exhibits a lower spatial resolution (*c.*0.25°), it has the advantage of a higher temporal resolution. We simulated vector control of *Ae. j. japonicus* by manipulating its mortality and reproduction rate during certain time frames for specific life stages (Additional file 1: Table S3). Every control scenario was performed with mortalities elevated by 50% and 80%, or in case of the oviposition scenario, reproduction rate was lowered by 50% and 80%. In our model, vector control measures are always introduced in the second year and population density of *Ae. j. japonicus* is calculated in the fifth year of the simulation run. Thus, vector control measures are applied in the simulations for four consecutive years. This ensures that population dynamics have reached an equilibrium state. The cumulative density in the fifth year of every cell is normalised by the highest population density in the default scenario, without any vector control measure. Instead of defining a specific month in which vector control measures should be applied, we decided to choose temperature limits that will provide a time frame of approximately 30 days (5–9 °C and 9–14 °C, Additional file 1: Table S3). This ensures that vector control measures will target similar stages in annual dynamics. Additionally, we applied scenarios with permanently elevated mortalities.

## Results

### Life history traits

Egg development was defined as a period of 10 days, regardless of temperature [55, 56]. Per capita loss rate during the egg stage differed between temperatures and a nonlinear function was fitted (see Additional file 1: Figure S2). Female fecundity was estimated by using a regression relating female wing lengths to fecundity [47]. We fitted a logistic function to the resulting temperature-dependent birth rate (Additional file 1: Figure S6). To obtain an estimate for population capacity, we rescaled mortality due to larval competition (Additional file 1: Figure S3c) to a linear function with complete mortality at 10^6^ individuals per grid cell. All parameters used in the model are summarised in Additional file 1: Table S2 and detailed in Text S2. Raw data are provided in Additional file 2.

### Current condition

Depending on local temperatures, the annual density dynamics in every life stage of *Ae. j. japonicus* can differ profoundly. While a stable population occurs in southwestern regions of Germany (e.g. Lahr, Fig. 1a), our prediction shows in other regions (e.g. Oberstdorf, Fig. 1b) that even though populations within the year of introduction might exhibit similar densities, the population will die out over the winter months. Thus, we conclude that at such locations no stable population can establish.

**Fig. 2:**
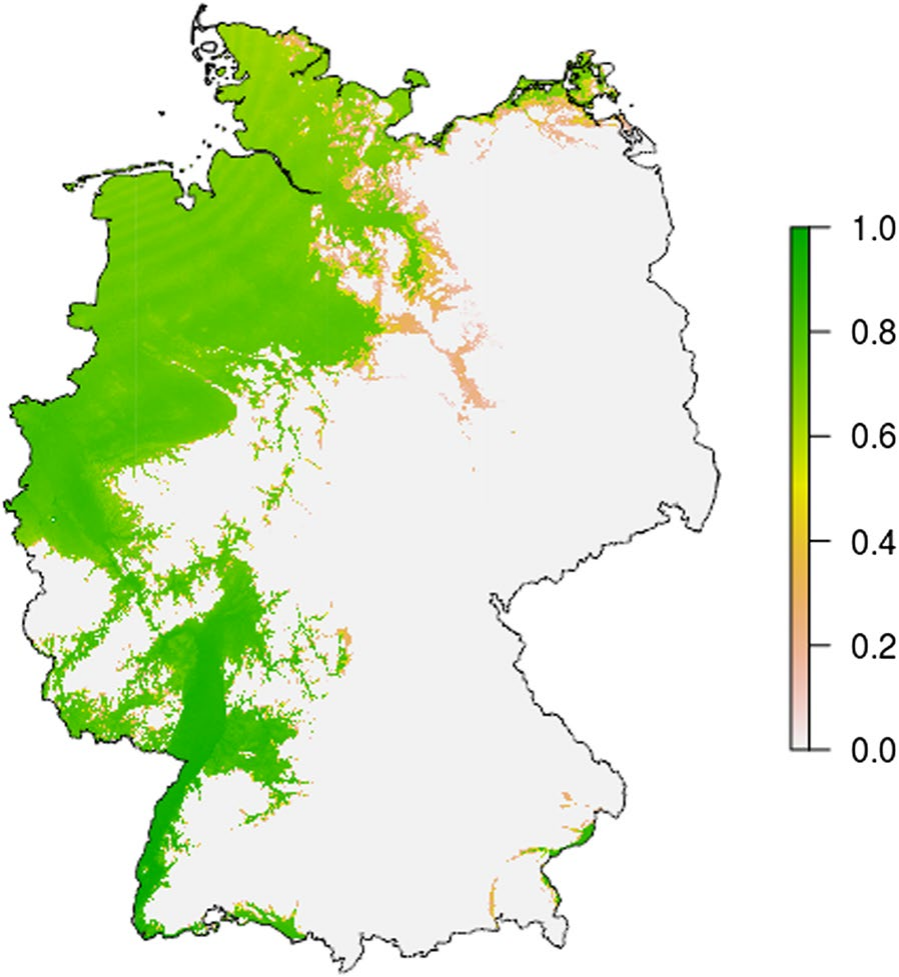
Modelled cumulative population density of *Aedes j. japonicus* larvae in Germany. Highest densities are observed in the south-west of Germany along the river Rhine. All values are normalised by the highest observed larval density. Temperature raster is based on the CHELSA dataset (1993–2013 [49, 50]), resolution 30′′

Performing this simulation for every cell in the CHELSA raster, then calculating and normalising the cumulative annual larval density shows population densities and stability in different regions (Fig. 2). Population density is expected to be highest along the River Rhine in the southwestern parts of Germany.

### Climate change

**Fig. 3:**
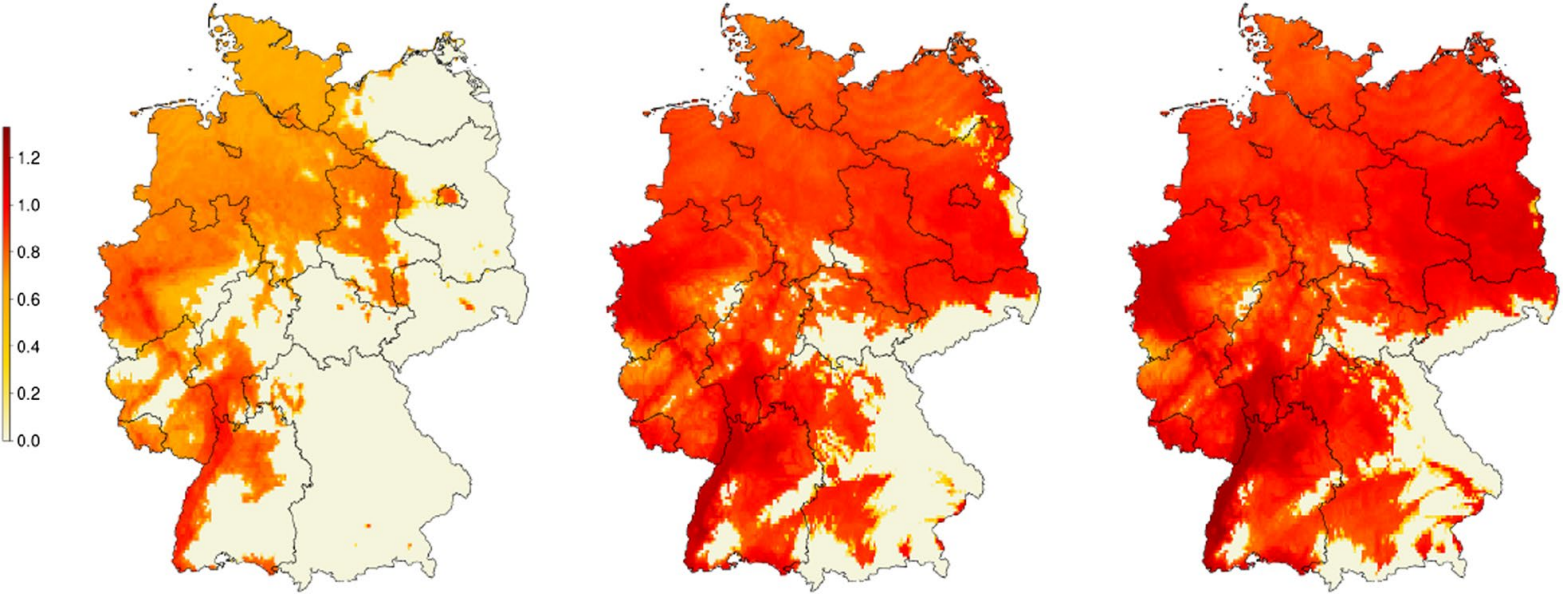
Modelled cumulative population density of *Aedes j. japonicus* larvae in Germany under climate change models. Scenarios current condition (left), CCSM4 RCP4.5 (middle), CCSM4 RCP8.5 (right), time period 2041–2060. Underlying raster layer is based on the CMIP 2.5’ models (approximately 4.5 km at equator), time period 2041–2060, from Worldclim database [51]. All larval densities are normalised by the highest observed larval density in the current condition scenario. Simulations ran for 5 years, densities were calculated in the fifth year

Climate change scenario CCSM4 RCP4.5 (2041–2060) is associated with a range expansion of stable population areas to the east (Fig. 3, middle panel). Regions in low mountain ranges (e.g. Harz, Rhön, Swabian Jura, etc.) still do not support the establishment of *Ae. j. japonicus*. Additionally, compared to the current scenario, population densities are expected to rise. Climate change scenario CCSM4 RCP8.5 (2041–2060) with higher emission values shows slightly further expansion of the invasive species and population densities up to 30% higher than in the current scenario (Fig. 3 right panel).

### Vector control measures

**Fig. 4:**
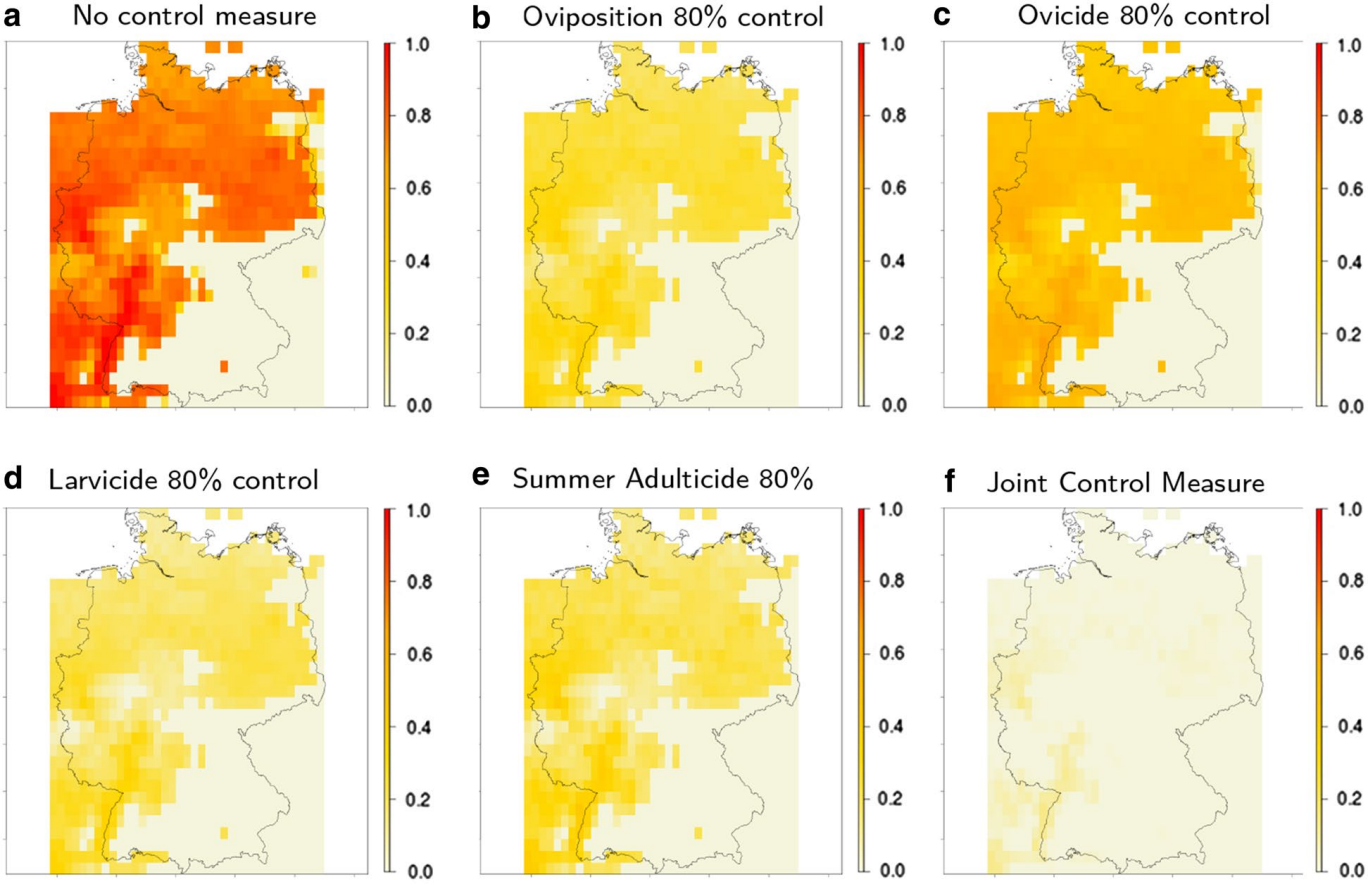
Modelled cumulative population density of *Aedes j. japonicus* larvae in Germany under different efficiency scenarios of vector control measures: **a** default scenario without any vector control measure; **b** constant control in oviposition, i.e. permanently reduced reproduction rate by 80%; **c** egg mortality raised permanently by 80%; **d** larval mortality raised permanently by 80%; **e** adult mortality raised by 80% during summer; **f** combination of constantly raised larval mortality and raised adult mortality during summer (both 50%). All values are normalised by the highest observed larval density. Temperature raster is based on ENSEMBLES dataset [52], resolution *c.*25 km

**Fig. 5:**
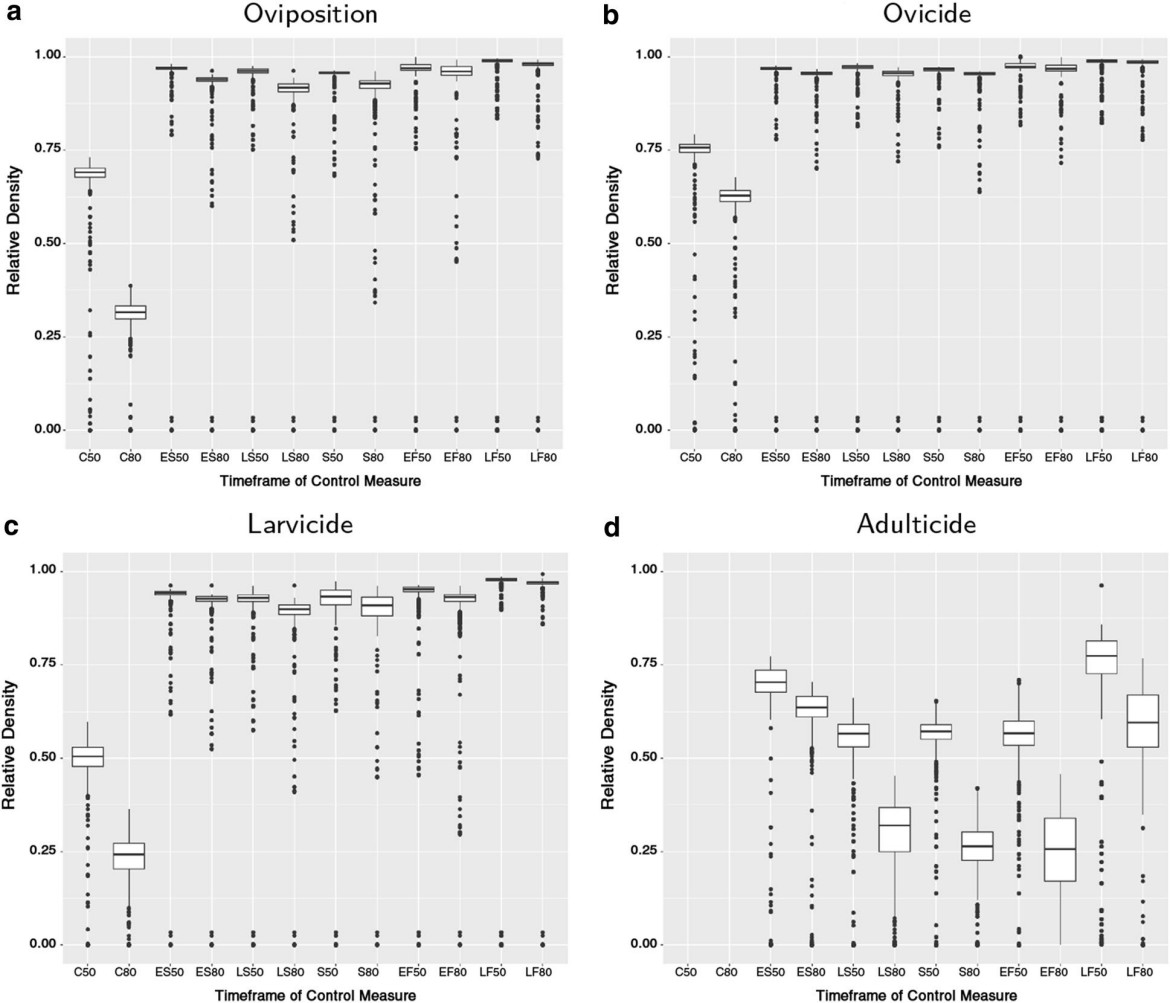
The mean reduction of population density overall efficiency scenarios of vector control measures. **a** Oviposition, targeting the reproduction. **b** Ovicide, targeting the egg stage. **c** Larvicide, targeting the larval stage. **d** Adulticide, targeting premature and adult stages. Timeframes: C, constant control measure; ES, early spring; LS, late spring; S, summer; EF, early fall; LF, late fall

The tested vector control measures yielded different levels of success in limiting or even eradicating the established populations. As can be seen in Figs. 4, 5, while every constant control measure has some effect on the population density, targeting the adult stage is the most successful control measure (mean reduction of 100%, Additional file 1: Figure S13a, b and Table S4), followed by application of larvicides (mean reduction of 76.8%), reduced oviposition (mean reduction of 69.2%), and heightened egg mortality (mean reduction of 39.7%, Fig. 5).

Control of the adult stage through elevated mortality yielded the best overall results. Comparing different timeframes show best results for control measures applied from late spring to early autumn. A scenario of constant larvicide application combined with summer adulticides (both 50% elevated mortalities) shows the potency of joint control measures (Fig. 4f).

A full account of the results of the different control scenarios is provided in Additional file 1: Text S4.

## Discussion

### Current condition

The findings of this study suggest that the annual population dynamics follows the expectations in a stage-structured population of ectotherms, as seen, e.g. in [39], with very low densities in the first half of the year and extreme population growth in the latter part. We observed a transition from discrete to overlapping generations, also called generation smearing. Larval density peaks in the second half of the year occur due to exponential population growth with limiting carrying capacity. The population ‘overshoots’ the maximum capacity, and density effects during the larval stage then lead to a population crash until larval density is again well below the maximum carrying capacity. Thus, these density peaks do not coincide with generation separation, an assumption supported by the fact that if larval competition is removed from the model, the resulting population dynamics during the second half of the year become an exponential curve (see Additional file 1: Figure S7). Reports on field populations support our prediction that generations can overlap, as different life stages are found simultaneously [57, 58].

The simulations showed that in many parts of Germany, it is possible for *Ae. j. japonicus* to successfully establish. Especially river valleys and plains in the southwest and west, as well as the lower regions of northern Germany seem to support multi-annual populations. Only regions in the southeast and east seem to be free from populations that last for longer than 1 year. On a side note, however, even in the southeast, we observe some ‘pockets’ for potential establishment of *Ae. j. japonicus* populations along the rivers Danube and Inn. This result might help to understand the reported population found in Upper Bavaria and Austria [19]. Still, the highest population densities are predicted along the rivers Rhine, Main and Neckar, which are in fact all regions where *Ae. j. japonicus* is already a well-known and prevalent organism [18]. Concurrently, the regions along the Rhine are among the most densely populated areas in Germany, which poses increased risk for public health. It becomes clear that the invasive organism has the potential to spread over large parts of the country and become a pest in many regions.

Note that in our model we did not implement migration from one cell of the raster to the neighboring. We chose this approach for several reasons: (i) while *stagePop* allows for migration terms, it is not meant to work in a spatial framework, meaning individual cells are independent from each other. (ii) Even though there are studies on active flight distance in mosquitoes [59], we would not be able to include anthropogenic factors, which are likely crucial in migration patterns. (iii) In this study we aimed to identify ‘high risk areas’ where survival and population growth is possible over multiple years without the aid of continuous immigration of new individuals.

### Climate change

Our climate change models predict that the population densities of *Ae. j. japonicus* will rise, and the species’ range will eventually cover all of Germany. As different climate data sets were used for current and future scenarios, bear in mind that Fig. 2 and 3 are not comparable. The current scenario relating to climate change can be found in Fig. 3. Interestingly, our result contradicts predictions of previously published species distribution models. Cunze et al. [60] argue, as *Ae. j. japonicus* is a temperate species, it will not survive higher temperatures during summer. However, our life-history data show that larval and pupal mortality rise only at 28 °C. This elevated mortality during a ‘heat wave’ would have an effect similar to the results of application of larvicides during summer (Additional file 1: Figure S12g, h). The diverging results between our model and Cunze et al. [60] are very likely due to the different approaches, ours being exclusively based on physiological data, while Cunze et al. [60] incorporate presence data and precipitation. Additionally, we used a different climate change projection (CCSM4). Our results suggest that a further spread of the species cannot be avoided if no control measures are taken to contain the population.

### Vector control measures

The different vector control measures show that oviposition deterrents, ovicides and larvicides show only little effect if applied short term. Larvicides show good results if applied continuously (Fig. 5c). Targeting oviposition, equivalent to reducing the reproduction rate, e.g. by means of SIT, shows slightly lower success than the application of larvicides (Figs. 4b, 5a). However, by far the most successful measure of control is the application of adulticides, potentially even purging the population completely. Unfortunately, many measures of adult control, such as chemical insecticides, lack the specificity of larvicides (e.g. Bti), or reproductive control (e.g. SIT), and are therefore questionable methods of control, risking unforeseen side effects on the environment. However, as seen in the joint control scenario, short-term adulticides in combination with constant larvicide application could be a decent compromise between efficiency and environmental protection (Fig. 4f).

We can hypothesise as to the reasons why apparently the adult stages are the most susceptible to control measures. By far the highest influence on mortality stems from larval competition, i.e. density effects in the larval stage. Potentially, the effect of control measures on early stages can be compensated due to the extreme overproduction of offspring. In part, this oddity in our model is due to the fact that while we have a decent idea about mortality in laboratory populations, factors such as predation or parasitism are not included in our modelling approach. On the other hand, our results show that overproduction of offspring can compensate many unforeseen hazards to the population.

We acknowledge that our approach to postulate scenarios with mortalities raised by, and reproduction lowered by 50 and 80% is very simplistic. Empirical studies (e.g. Fonseca et al. [61]) on the success of different vector control measures report success rates between 40 and 75%, which we used as a guideline. However, as Fonseca et al. [61] state, success rates heavily depend on location, urban areas being easier to control than forests. Other factors that could not be reliably modelled include the quality of the implemented control, e.g. specific policies (such as education campaigns) concerning vector control measures in different regions.

### Comparison to species distribution models

It has to be noted, that the presented method is not a species distribution model, and disregards actual presence/absence data. Melaun et al. [62] provided such a model based on occurrence and environmental data. Most occurrence data of *Ae. j. japonicus* (85.7%) fits in our model, especially in southwestern Germany; however, 14.3% of observations lie outside our predicted range (Additional file 1: Figure S9, [17, 19, 48, 62–69]). There are several possible reasons why our model fails to reproduce these data points. As discussed in Tjaden et al. [70] the different methods and used parameters in correlative (e.g. species distribution models) and mechanistic models (our model) can lead to very different outcomes. Efforts to combine aspects of both approaches could help to overcome this. As mentioned earlier, we only identify core regions of stable populations. Repeated introduction or migration, which is most likely a central factor in the spread of many invasive mosquitoes [16, 68], is not incorporated in our model. Human interaction, be it through inadvertent dislocation or the provision of shelter or suitable microhabitats during freezing events in winter or heat waves during summer, is also not modelled in this approach. Additionally, the life-cycle parameters used in our model were gathered on a population from Lahr, an especially mild region in Germany. It is quite possible that due to rapid thermal adaptation [71] and the relatively high number of generations since their introduction [72], some populations have already adapted to their local environments. Alternatively, if *Ae. j. japonicus* was introduced to Europe from multiple origins, these ‘strains’ of populations from different source populations could exhibit adaptations to various degree. Finally, our model focuses on the organism’s dependency on temperature. Although this factor is central in the development and survival of the individual, it is by far not the only factor influencing it. Availability of water sources suitable as larval breeding sites also plays an important role in the establishment of stable populations. Furthermore, changes in day length significantly impact the development of *Ae. j. japonicus* [9], potentially responsible for inducing diapause and synchronizing the start of population growth at the beginning of the year.

One missing parameter in our model is the influence of precipitation. Studies on *Ae. albopictus* are not conclusive [73] whether rainfall has a positive [74], negative [75], or no effect [76] on population growth in container-inhabiting mosquitoes. Thus, we chose to formulate a ‘worst case scenario’, in which individuals will always find a body of water, regardless if it is naturally occurring or of human origin. However, prolonged drought periods like in 2018 have the potential to significantly reduce available breeding habitats with negative consequences for population density.

One should always bear in mind the assertion that all models are wrong, but some are useful [77] holds true for the presented method. This becomes obvious, when comparing our predictions with Melaun et al. [62]. While some conclusions concur, such as prediction of occurrence along the River Rhine, other predictions in the northwest and southeast of Germany diverge between the models.

## Conclusions

The identification of regions with stable populations offers the possibility to register ‘high risk areas’, where the population density during summer is expected to be especially high and which might function as refugia during the winter months. We recommend that these regions should be the preferred targets of any control measure. Not only could this alleviate the nuisance and health risk for the local human population, but minimise further spread of the species into neighbouring regions. However, as the actual range of the species exceeds the predicted area, further measures to educate the general public could be crucial in the containment of the invader.

## Additional files

**Additional file 1: Text S1.** R-Scripts used for simulations. **Text S1.1.** Script for calling and parallelising stagePop function. **Text S1.2.** Script for R-package stagePop. **Text S1.3.** Script for visualization. **Text S2.** Detailed parameter elicitation. **Text S2.1.** A linear model utilizing a sine-cosine curve is fitted to temperature data. **Figure S1.** Example for daily mean temperature and fitted curve. **Text S2.2.** Development during egg stage. **Figure S2.** Through-stage mortality in eggs. **Text S2.3.** Parameters for larval stages. **Figure S3.** Parameters for larval stage. **Text S2.4.** Parameters for pupal stage. **Figure S4.** Parameters for pupal stage. Text **S2.5.** Daily mortality rate in premature and adult stage. **Figure S5.** Daily mortality rate in premature and adult stage. **Text S2.6.** Approximation of birth rate through wing length. **Text S2.7.** Parameter Summary. **Table S1.** Mean wing lengths in 14 temperatures and estimated female fecundity. **Figure S6.** Number of offspring per female. **Table S2.** List of parameters used in population dynamics model. **Text S3.** Population Density and Continuity. **Figure S7.** Population Dynamics if no larval competition is applied. **Figure S8.** Cumulative larval density without any control measure. **Figure S9.:** Our model compared with published occurrence data [17–19, 48, 62–65, 67, 68]. **Text S4.** Control Measures.  **Table S3.** Different scenarios in pest control measures. **Figure S10.** Density of larvae if control measure targets oviposition. **Figure S11.** Density of larvae if control measure targets egg mortality. **Figure S12.** Density of larvae if control measure targets larval mortality. **Figure S13.** Density of larvae if control measure targets adult mortality. **Figure S14.** Combination of constant larval control (50%) and summer adult control (50%). **Table S4.** Results of control measures. **Text S5.** Discussion of winter mortality. **Figure S15.** Annual population density dynamics of larvae in scenarios with different winter mortality. **Figure S16.** Density of larvae in scenarios with different winter mortality.

**Additional file 2: Table S5.** Raw data of the hatching experiment.

## Abbreviations

Bti: *Bacillus thuringiensis israelensis*
CCSM: Community climate system model
CMIP: Coupled model intercomparison project
RCP: Representative concentration pathway
RNAi: Ribonucleic acid interference
SIT: Sterile-insect techniques
