# Supplementary material for: Modelling seasonal dynamics, population stability, and pest control in Aedes japonicus japonicus (Diptera: Culicidae)
Source: Parasit Vectors. 2019 Mar 25;12:142. doi: 10.1186/s13071-019-3366-2 (PMC6434845; doi:10.1186/s13071-019-3366-2)
Supplement: Supplementary file 1 — Additional file 1: Text S1. R-Scripts used for simulations. Text S1.1. Script for calling and parallelising stagePop function. Text S1.2. Script for R-package stagePop. Text S1.3. Script for visualization. Text S2. Detailed parameter elicitation. Text S2.1. A linear model utilizing a sine-cosine curve is fitted to temperature data. Figure S1. Example for daily mean temperature and fitted curve. Text S2.2. Development during egg stage. Figure S2. Through-stage mortality in eggs. Text S2.3. Parameters for larval stages. Figure S3. Parameters for larval stage. Text S2.4. Parameters for pupal stage. Figure S4. Parameters for pupal stage. Text S2.5. Daily mortality rate in premature and adult stage. Figure S5. Daily mortality rate in premature and adult stage. Text S2.6. Approximation of birth rate through wing length. Text S2.7. Parameter Summary. Table S1. Mean wing lengths in 14 temperatures and estimated female fecundity. Figure S6. Number of offspring per female. Table S2. List of parameters used in population dynamics model. Text S3. Population Density and Continuity. Figure S7. Population Dynamics if no larval competition is applied. Figure S8. Cumulative larval density without any control measure. Figure S9.: Our model compared with published occurrence data [17–19, 48, 62–65, 67, 68]. Text S4. Control Measures. Table S3. Different scenarios in pest control measures. Figure S10. Density of larvae if control measure targets oviposition. Figure S11. Density of larvae if control measure targets egg mortality. Figure S12. Density of larvae if control measure targets larval mortality. Figure S13. Density of larvae if control measure targets adult mortality. Figure S14. Combination of constant larval control (50%) and summer adult control (50%). Table S4. Results of control measures. Text S5. Discussion of winter mortality. Figure S15. Annual population density dynamics of larvae in scenarios with different winter mortality. Figure S16. Density of larvae in scenarios w [file 13071_2019_3366_MOESM1_ESM.pdf]

Modelling seasonal dynamics, population stability, and pest control in *Aedes japonicus japonicus* (Diptera: Culicidae)

Additional file 1

Andreas Wieser[1,2,3], Friederike Reuss[1,4], Aidin Niamir[1], Ruth Müller[5,6], Robert B. O'Hara[1,3] and Markus Pfenninger[1,2]

March 14, 2019

Contents

1 Text S1 – R-Scripts used for simulations 2

1.1 Text S1.1 – Script for calling and parallelising stagePop function 3

1.2 Text S1.2 – Script for R-package StagePop 5

1.3 Text S1.3 – Script for visualization 13

2 Text S2 – Detailed Parameter elicitation 14

2.1 Text S2.1 – A linear model utilizing a sine-cosine curve is fitted to temperature data 14

2.2 Text S2.2 – Development during egg stage 15

2.3 Text S2.3 – Parameters for larval stages 16

2.4 Text S2.4 – Parameters for pupal stage 17

2.5 Text S2.5 – Daily mortality rate in premature and adult stage 18

2.6 Text S2.6 – Approximation of birth rate through wing length 19

2.7 Text S2.7 – Parameter Summary 20

3 Text S3 – Population Density and Continuity 21

4 Text S4 – Control Measures 23

5 Text S5 – Discussion of Winter Mortality 29

1 Text S1 – R-Scripts used for simulations

We model five life stage of *Aedes japonicus japonicus*: egg, larva, pupa, sexually immature adults before the intake of a blood-meal ("premature") and adults. For each life stage we obtain a daily development rate and through-stage mortality rate. These factors tend to be temperature dependent, however, for some parameters only estimates could be obtained (Kettle and Nutter, 2015; Gurney and Nisbet, 1998). Below, we provide the `R`-codes to our models. It can be adapted to accomodate different input datasets, i.e. daily and monthly temperature data and all control measures are included in commented sections of the scripts.

1 Senckenberg Biodiversity and Climate Research Centre, Senckenberganlage 25, 60325 Frankfurt am Main, Germany
2 Institute of Organismic and Molecular Evolution (iOME), Johannes Gutenberg University, Gresemundweg 2, 55128 Mainz, Germany
3 Centre for Biodiversity Dynamics, and Department of Mathematical Sciences, Norwegian University of Science and Technology NTNU, Sentralbygg 2, Gløshaugen, 7491 Trondheim, Norway.
4 Institute for Ecology, Evolution and Diversity, Faculty of Biological Sciences, Goethe University, Max-von-Laue-Straße 9, 60438 Frankfurt am Main, Germany
5 Faculty of Medicine, Institute of Occupational Medicine, Social Medicine and Environmental Medicine, Goethe University, Theodor-Stern-Kai 7, 60590 Frankfurt am Main, Germany
6 Unit of Entomology, Institute of Tropical Medicine, Nationalenstraat 155, 2000 Antwerp, Belgium

1.1 Text S1.1 – Script for calling and parallelising stagePop function

```
#Script to call and parallelise stagepop

#Andreas Wieser 09.04.2018

#Input: It is necessary to provide temperature data, e.g. in a *
    .RDS-file

setwd("/home/user/your/path")

#args should be the corresponding stagepop_script.R
args <- commandArgs(TRUE)

source(args[1])

#load temperature data from data.RDS
tempdata <- readRDS("data.RDS")

transformed <- t(tempdata)

#We choose a period of 20 years from the data
Time <- c(1:7305)
removed <- transformed[ , ! apply( transformed , 2 , function(x)
     any(is.na(x)) ) ]
truncated <- removed[{24716-7305-242}:{24716-243},]

#setup for automation
library(R.utils)
library(snow)

#if stagepop simulation fails , it can run indefinitely. We
    suggest to counter this with a timeout.
interruptor = function(FUN,args, time.limit){
  results <- NULL
  results <- withTimeout({FUN(args)},timeout=time.limit,
     onTimeout="silent")
  if(is.null(results)){
    results <- 0
  }
  return(results)
}

#setup cluster
cl <- makeCluster(getOption("cl.cores", 120))
clusterExport(cl, list("interruptor","funForInput","withTimeout"
    ,"Time"))
res <- (parCapply(cl,truncated,function(x) interruptor(
    funForInput,x,time.limit=1000)))
stopCluster(cl)

#treat output
```

```
resnorm <- res/max(res)
resnorm[resnorm<0.001]<-0

curCond <- data.frame(removed[1,],res,resnorm)
colnames(curCond) <- c("ID","absolute","density")

saveRDS(curCond, file=paste(args[1],".rds", sep=""))
```

1.2 Text S1.2 – Script for R-package StagePop

```
#StagePop Script for Annual Dynamics of Aedes japonicus
    japonicus

#Andreas Wieser 09.04.2018

#Objective:
#This script offers a complete summary of the population
    dynamics models including all control measures (commented out
     for convenience).

#transformed into function
#requirements: stagepop (R-package) and dependencies
#input: a dataframe with daily mean temperature and dates:
# temp        date
# 0.00        2000-01-31 (%Y%m%d)


funForInput <-  function(localtemp){

  #fitting the temperature curve to data from localtemp
  xc<-cos(2*pi*Time/365)
  xs<-sin(2*pi*Time/365)
  fit.lm <- lm(localtemp~xc+xs, na.action=na.exclude)
  intercept <- coef(fit.lm)[1]
  cosineFactor <- coef(fit.lm)[2]
  sineFactor <- coef(fit.lm)[3]

  #access the fitted series (for plotting)
  fit <- fitted(fit.lm)

  #find predictions for time series
  pred <- predict(fit.lm, newdata=data.frame(Time=Time))

  #this part looks for the lowest values. This is used to find
      the threshold for freezing events. At least one freezing
      event of four days is assumed for every cell.
  N<-4
  ndx=order(fitted(fit.lm)[1:365])
  thresholdIndex=ndx[N]
  threshold= fitted(fit.lm)[thresholdIndex]

  #all values of the year listed
  allyear=fitted(fit.lm)[1:365]

#StagePop
#Model for Aedes japonicus including 5 stages, freezing event,
    density dependency in larval stage
library(stagePop)
library(deSolve)

#Settings for stagepop: both variants work.
#solver.options=list(DDEsolver='deSolve', atol=1e-14,rtol=1e-16,
```

```
    hbsize=1e9)
solver.options=list(DDEsolver='PBS', tol=1e-8,hbsize=1e4,dt=0.01)

#Airtemp
#Temperature function based on data from localtemp
tempAir=function(time){
  xc<-cos(2*pi*time/365)
  xs<-sin(2*pi*time/365)
  A= intercept + cosineFactor *xc + sineFactor *xs
  return(A)}

##Here we predefine the development functions. They are called
    again later in the script.
#Development function for larvae. Very slow under 7 degrees,
    otherwise 10 days.
eggFunc=function(A){
  if(A<7){v=100}
  else{v=10}
  return(v)
}

#Time to maturation of adults. Considered to be the same as
    average time until blood-meal was obtained. Always 14 days.
adFunc=function(A){
  v=14
  return(v)
}

#Development function of larvae
tauFunc=function(A){
  u=0.01507*exp(0.07507*A)
  v=1/u
  return(v)}

#Development function for pupae
tauPupae=function(A){
  u=0.04137*exp(0.09234*A)
  v=1/u
  return(v)
}

##This section defines all functions for stagepop
varDurEnvFunctions<-list(
  #birth rate
  reproFunc=function(x,time,species,strain){
    A=tempAir(time)
    #Adults will not reproduce below 5 degrees.
    if(A<5){reprod=x$Aedes['adults',1]}
    else{
        #Reproduction depending on temperature.
        reprod=(115.278/(1+exp((29.121-A)/-5.846)))*x$Aedes['
            adults',1]}
        #Scenario "Reduced Oviposition": Manipulation of
            reproduction as control measure
```

```
        #Constant Control
          #reprod=(115.278/(1+exp((29.121-A)/-5.846)))*x$Aedes
              ['adults',1]*0.5}
        #Early Spring
          #if(time>365){
            #if(A>5 & A<9 & tempAir(time)>tempAir({time-1})){
              #reprod=(115.278/(1+exp((29.121-A)/-5.846)))*x$
                  Aedes['adults',1]*0.5
            #}
          #}
        #Late Spring
          #if(time>365){
            #if(A>9 & A<14 & tempAir(time)>tempAir({time-1})){
              #reprod=(115.278/(1+exp((29.121-A)/-5.846)))*x$
                  Aedes['adults',1]*0.5
            #}
          #}
        #Summer
          #if(time>365){
            #if(A>tempAir(ndx[335])){
              #reprod=(115.278/(1+exp((29.121-A)/-5.846)))*x$
                  Aedes['adults',1]*0.5
            #}
          #}
        #Early Fall
          #if(time>365){
            #if(A>9 & A<14 & tempAir(time)<tempAir({time-1})){
              #reprod=(115.278/(1+exp((29.121-A)/-5.846)))*x$
                  Aedes['adults',1]*0.5
            #}
          #}
        #Late Fall
          #if(time>365){
            #if(A>5 & A<9 & tempAir(time)<tempAir({time-1})){
              #reprod=(115.278/(1+exp((29.121-A)/-5.846)))*x$
                  Aedes['adults',1]*0.5
            #}
          #}

  return(reprod)},

#mortality, stage specific
deathFunc=function(stage,x,time,species,strain){
  A=tempAir(time)

  #egg mortality, temp dependent;
  gamma=exp(-0.8352-0.05423*A)

  #Scenario "Egg Mortality": Manipulation of egg mortality as
      control measure.
    #Constant control
      #if(time>365){
        #gamma=.5+exp(-0.8352-0.05423*A)
      #}
```

```
        #else{gamma=exp(-0.8352-0.05423*A)
      #}
    #Early Spring
      #if(time>365){
        #if(A>5 & A<9 & tempAir(time)>tempAir({time-1})){
          #gamma=exp(-0.8352-0.05423*A)+0.5
        #}
      #}
    #Late Spring
      #if(time>365){
        #if(A>9 & A<14 & tempAir(time)>tempAir({time-1})){
          #gamma=exp(-0.8352-0.05423*A)+0.5
        #}
      #}
    #Summer
      #if(time>365){
        #if(A>tempAir(ndx[335])){
          #gamma=exp(-0.8352-0.05423*A)+0.5
        #}
      #}
    #Early Fall
      #if(time>365){
        #if(A>9 & A<14 & tempAir(time)<tempAir({time-1})){
          #gamma=exp(-0.8352-0.05423*A)+0.5
        #}
      #}
    #Late Fall
      #if(time>365){
        #if(A>5 & A<9 & tempAir(time)<tempAir({time-1})){
          #gamma=exp(-0.8352-0.05423*A)+0.5
        #}
      #}

  #larval mortality, temp and density dependent;
  delta=(0.92964*exp(-0.07226*A)+ (0.0764 + 0.8764 * (x$Aedes[
      'larvae',]/1e6)))

  #Scenario "Larval Mortality": Manipulation of larval
      mortality as control measure.
    #Constant control
      #if(time>365){
        #delta=0.5+(0.92964*exp(-0.07226*A)+ (0.0764 + 0.8764
            * (x$Aedes['larvae',]/1e6)))
      #}
        #else{delta=(0.92964*exp(-0.07226*A)+ (0.0764 + 0.8764
             * (x$Aedes['larvae',]/1e6)))}
  #Early Spring
      #if(time>365){
        #if(A>5 & A<9 & tempAir(time)>tempAir({time-1})){
          #delta=0.5+(0.92964*exp(-0.07226*A)+ (0.0764 +
              0.8764 * (x$Aedes['larvae',]/1e6)))
        #}
      #}
  #Late Spring
```

```
    #if(time>365){
      #if(A>9 & A<14 & tempAir(time)>tempAir({time-1})){
        #delta=0.5+(0.92964*exp(-0.07226*A)+ (0.0764 +
            0.8764 * (x$Aedes['larvae',]/1e6)))
      #}
    #}
#Summer
    #if(time>365){
      #if(A>tempAir(ndx[335])){
        #delta=0.5+(0.92964*exp(-0.07226*A)+ (0.0764 +
            0.8764 * (x$Aedes['larvae',]/1e6)))
      #}
    #}
#Early Fall
    #if(time>365){
      #if(A>9 & A<14 & tempAir(time)<tempAir({time-1})){
        #delta=0.5+(0.92964*exp(-0.07226*A)+ (0.0764 +
            0.8764 * (x$Aedes['larvae',]/1e6)))
      #}
    #}
#Late Fall
    #if(time>365){
      #if(A>5 & A<9 & tempAir(time)<tempAir({time-1})){
        #delta=0.5+(0.92964*exp(-0.07226*A)+ (0.0764 +
            0.8764 * (x$Aedes['larvae',]/1e6)))
      #}
    #}

#pupal mortality, temp dependent
epsilon=(0.23251*exp(-0.06276*A) + 1.69e-13*exp(9.23e-1*A))

#adult (both premature and mature) mortality, temp dependent
omega=0.03747*exp(0.06113*A)

#Scenario "Adult Mortality": Manipulation of adult mortality
    as control measure.
  #Constant control
    #if(time>365){
    #  omega=0.5+0.03747*exp(0.06113*A)
    #}
    #else{omega=0.03747*exp(0.06113*A)
    #}
  #Early Spring
    #if(time>365){
      #if(A>5 & A<9 & tempAir(time)>tempAir({time-1})){
        #omega=0.5+0.03747*exp(0.06113*A)
      #}
    #}
  #Late Spring
    #if(time>365){
      #if(A>9 & A<14 & tempAir(time)>tempAir({time-1})){
        #omega=0.5+0.03747*exp(0.06113*A)
      #}
    #}
```

```
    #Summer
      #if(time>365){
        #if(A>tempAir(ndx[335])){
          #omega=0.5+0.03747*exp(0.06113*A)
        #}
      #}
    #Early Fall
      #if(time>365){
        #if(A>9 & A<14 & tempAir(time)<tempAir({time-1})){
          #omega=0.5+0.03747*exp(0.06113*A)
        #}
      #}
    #Late Fall
      #if(time>365){
        #if(A>9 & A<14 & tempAir(time)<tempAir({time-1})){
          #omega=0.5+0.03747*exp(0.06113*A)
        #}
      #}

  #after year 1, if temperature falls below 0 degrees,
      mortality of larval, pupal and adult stages is raised to
      1.
  if(time>365 & any(allyear<0) & A<0){
    delta=1
    epsilon=1

  }

  #if mean temp function does not drop below 0 degrees, we
      create an artificial freezing event, once a year for four
       days.
  if(time>365 & A<=threshold){
    delta=1
    epsilon=1

  }

  a=c(gamma,delta,epsilon,omega,omega)
  v=a[stage]
  return(max(0,v))
},

#Development functions for all stages
devel Func=function(stage,x,time,species,strain){
  A=tempAir(time)
  #egg development
  if(stage==1){v=1/eggFunc(A)}
  #larvae, development very slow below 7 degrees, above it
      follows tau function
  if(stage==2){if(A<7){v=0.1*1/tauFunc(A)}
          else{v=1/tauFunc(A)}}
  #pupae
  if(stage==3){v=1/tauPupae(A)}
  #adults
```

```
    if(stage==4){v=1/adFunc(A)}

    return(v)
  },

#starting conditions.
  durationFunc=function(stage,x,time,species,strain){
    if(time==0){
      A=tempAir(time)
      if(stage==1){v=10}
      if(stage==2){v=tauFunc(A)}
      if(stage==3){v=tauPupae(A)}
      if(stage==4){v=14}

    }
    return(v)
  },

#immigration serves as starting condition.
  immigrationFunc=function(stage,x,time,species,strain){
    v=0
    if(stage==5){if(time>=0 & time<=0.1){v=1}}
    return(v)
  },

#emigration. ignored in this model.
  emigrationFunc=function(stage,x,time,species,strain){
    return(0)}
)

#duration of simulation. We recommend running the simulations
    for at least 2-3 years until stable conditions are reached.
dur <- 365*5 +2

#model conditions. 1 species, 5 stages, density dependency and
    changing development.
modelOutput=popModel(
  numSpecies=1,numStages=5,
  timeDependLoss=TRUE, timeDependDuration=TRUE,
  ICs=list(matrix(0,nrow=5,ncol=1)),
  timeVec=seq(0,dur,1),
  solverOptions=solver.options,
  rateFunctions=varDurEnvFunctions,
  plotFigs = FALSE,
  stageNames=list(c('egg','larvae','pupae','premature','adults')
      ),
  speciesNames=c('Aedes'))

#just for plotting, saves plot in working directory
#z <- seq(as.Date("01/01/0001", format = "%d/%m/%Y"), by = "days
    ", length = dur)

#svg(filename="plot.svg")
```

```
#par(mar = c(5,5,2,5))
#plot(z,modelOutput[1:dur,3], type="l", col="red3", ylab="Larval
    Density [Individuals]", xlab="Time [in years]",main=
    expression(paste("Annual Dynamics localtemp")))

#par(new = TRUE)
#plot(modelOutput[,5], type="l",axes=F, xlab=NA, ylab=NA)
#axis(side = 4)
#mtext(side = 4, line = 3, 'Adult Density [Individuals]')
#legend("topleft",
#        legend=c("Larvae", "Adults"),
#        lty=c(1,1), col=c("red3", "black"))

#dev.off()

#output: cumulative annual density of larvae
return(sum(modelOutput[{dur-365}:dur,3]))
  }
```

1.3 Text S1.3 – Script for visualization

```
library(raster)
library(RColorBrewer)
setwd("/home/user/your/path")

#args should be stagepop output (*.rds)
args <- commandArgs(TRUE)

# Raster template based on the Temp data
#r<-raster("tg_0.25deg_DE.grd")
r<-raster(nrow=31, ncol=37, xmn=5.75, xmx=15, ymn=47.25, ymx=55)
crs(r)<-"+proj=longlat +datum=WGS84 +ellps=WGS84 +towgs84=0,0,0 
    "

# Polygone Germany
de_border<-readRDS("DEU_adm0.rds")

# Inputs from stagepop
larval_density_abs <-readRDS("norm.R.rds")
larval_density <-readRDS(args[1])
larval_density$abscale <- larval_density$absolute/max(larval_
    density_abs$absolute)


# Assign cell values
for (i in larval_density$ID)
  {
  r[i]<-larval_density$abscale[larval_density$ID==i]
}

writeRaster(r,"larval_density_01.img",format="HFA", overwrite=
    TRUE)

# Plots
r<-raster("larval_density_01.img")
my.palette <- brewer.pal(n = 9, name = "YlOrRd")
png(filename = paste(args[1],".png", sep=""),width = 1000,
    height = 1000)
plot(r, col = my.palette, zlim=c(0,1))
plot(de_border,add=T)
dev.off()
```

2 Text S2 – Detailed Parameter elicitation

2.1 Text S2.1 – A linear model utilizing a sine-cosine curve is fitted to temperature data

A linear model utilizing a sine-cosine curve is fitted to temperature data from every location (e.g. Figure S1). The temperature function is then defined as

$$y = I + a \cdot \cos \frac{2\pi t}{d} + b \cdot \sin \frac{2\pi t}{d}, \tag{1}$$

where I is the intercept, a denotes the cosine cofactor, b denotes the sine cofactor, and t denotes time in days, d denotes temporal resolution, i.e. $d = 12$ for monthly data (CHELSA, Karger et al. (2017), Wordlclim, Hijmans et al. (2005)), $d = 365$ for daily data (ENSEMBLES, Haylock et al. (2008).

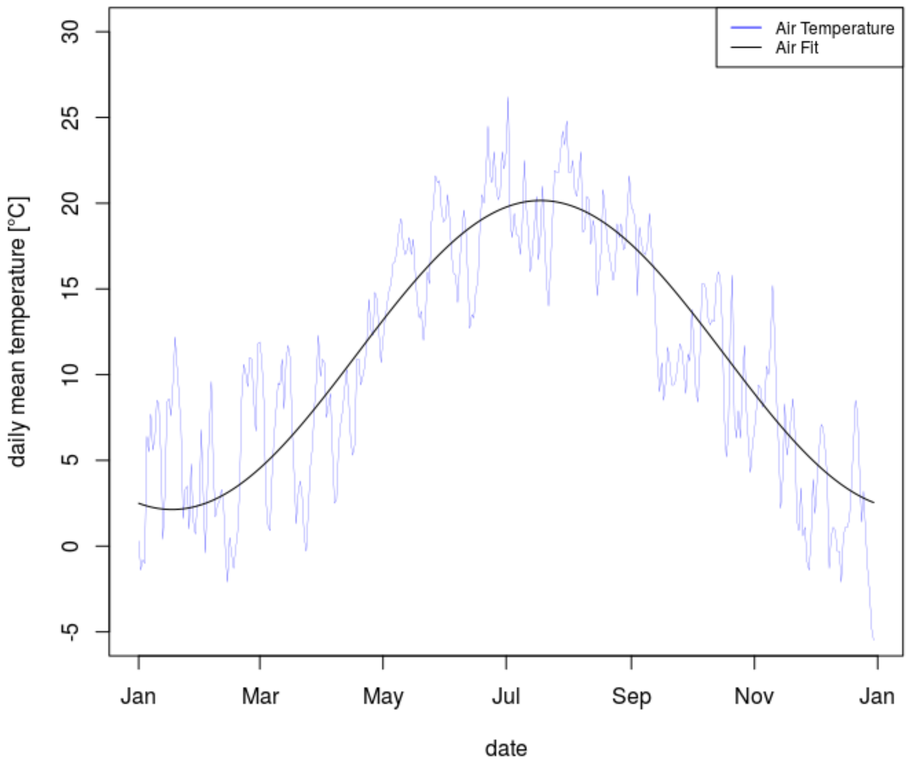

Figure S1 – Example for daily mean temperature and fitted curve. Temperature data from Lahr (Schwarzwald, Germany).

2.2 Text S2.2 – Development during egg stage

Development during egg stage is considered constant and temperature independent at 10 days. Through-stage mortality (Figure S2) was obtained for four temperatures and a decay curve was fitted. The equation for the fitted curve is given by $y = \exp^{(-0.8352-0.05423 \cdot t)}$.

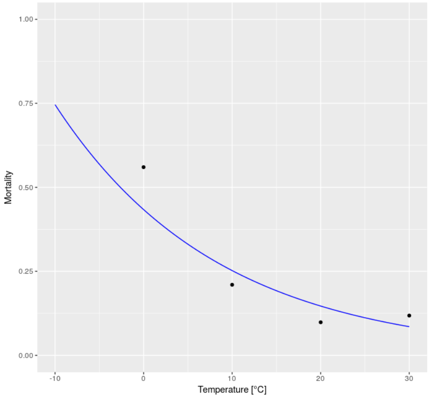

Figure S2 – Through-stage mortality in eggs.

2.3 Text S2.3 – Parameters for larval stages

Larval development is temperature dependent and defined as $y = 0.01831 \cdot \exp^{0.06559 \cdot t}$. A nonlinear model was used for fitting even though a linear fit would have been more appropriate (Figure S3). We chose a nonlinear fit to ease later model development. Larval mortality is high at temperatures lower than 10 °C and higher than 28 °C, thus we fit two different functions to the data (Figure S3). For temperatures up to 28 °C we use $y = 0.92964 \cdot \exp^{-0.07226 \cdot t}$, while for temperatures higher than 28 °C $y = 10^{-9} \cdot \exp^{\frac{0.9}{\log(0.22)^{-1}}}$ proved acceptable. During larval stage density dependent competition is another factor to be considered. We assume a linear relation as shown in Figure S3 and described by $y = 0.0764 + 0.004382 \cdot x$, where x denotes the density of individuals.

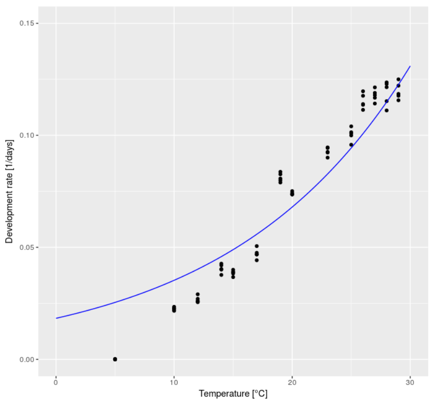

Daily development rate

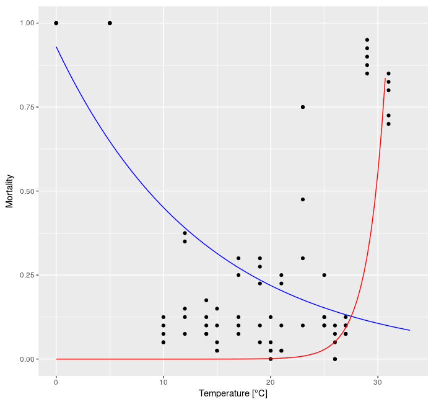

Through-stage mortality rate

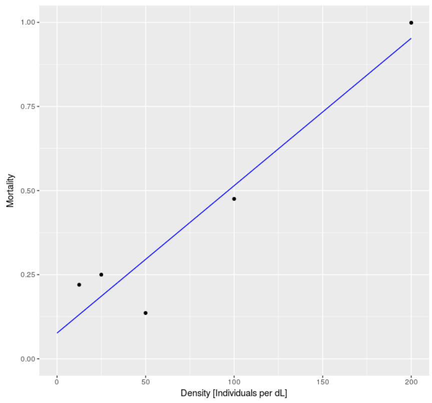

Density effect on mortality.

Figure S3 – Parameters for larval stage.

2.4 Text S2.4 – Parameters for pupal stage

Pupal development (Figure S4) is modelled by $y = 0.04137 \cdot \exp^{0.09234 \cdot t}$, mortality in low temperatures is defined as $y = 0.23251 \cdot \exp^{-0.06276 \cdot t}$, and as in larval stage mortality for high temperatures is included as well.

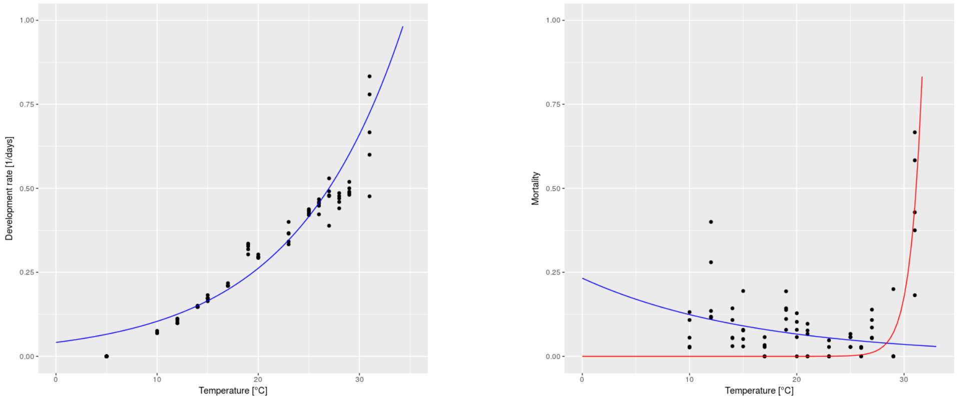

Daily development rate

Through-stage mortality rate

Figure S4 – Parameters for pupal stage.

2.5 Text S2.5 – Daily mortality rate in premature and adult stage

We assume a constant duration of 14 days until adults matured (blood-meal, mating). Adult mortality is modelled as $y = 0.03747 \cdot \exp^{0.06113 \cdot t}$.

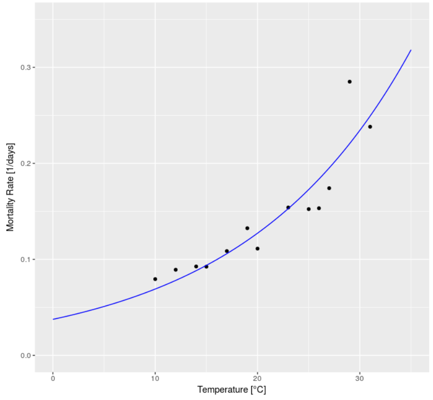

Figure S5 – Daily mortality rate in premature and adult stage.

2.6 Text S2.6 – Approximation of birth rate through wing length

As described in Armistead et al. (2008), number of offspring correlates with wing length of females as follows: $y = 53.078 \cdot x - 113.91$, where x denotes mean female wing length [in mm] and y denotes eggs per female (see Table S1). Thus, we model a logistic fit $y = \frac{115.278}{1+\exp\frac{29.121-x}{-5.846}}$ (see Fig. S6).

Table S1 – Mean wing lengths in 14 temperatures and estimated female fecundity.

**temperature**	x	y
10	4.183874	108.2
12	4.249405	111.6
14	4.157696	106.8
15	4.260843	112.2
17	3.97515	97.1
19	4.013494	99.1
20	3.926066	94.5
23	3.663847	80.6
25	3.692363	82.1
26	3.494686	71.6
27	3.41604	67.4
28	3.434567	68.4
29	3.182838	55.0
31	3.039693	47.4

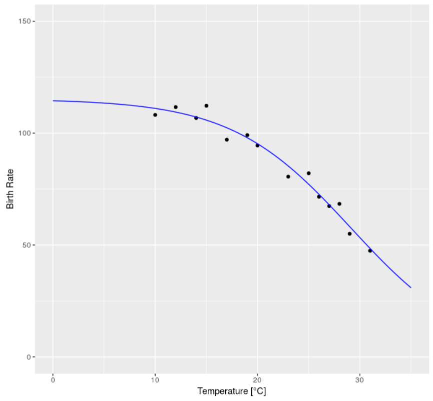

Figure S6 – Number of offspring per female.

2.7 Text S2.7 – Parameter Summary

Table S2 – List of parameters used in population dynamics model. Parameters are input in the following equations: for (a) $y = \alpha \cdot \exp(b + c \cdot t)$, for (c) $y = m + n \cdot x$, for (d) $y = \frac{\phi_1}{1+\exp\frac{\phi_2 - t}{\phi_3}}$; t corresponds to time in days, x equates to number of individuals. For (b) a constant duration in days was assumed.

**(a)**	α	b	c
Egg Mortality*	1	-0.054	-0.835
Larval Mortality**	0.930	0	-0.072
Larval Development	0.018	0	0.0656
Pupal Mortality**	0.233	0	-0.063
Pupal Development	0.041	0	0.092
Premature Adult & Adult Mortality	0.037	0	0.061
**(b)**	duration		
Egg Development	10		
Premature Adult Development	14		
**(c)**	m	n	
Larval Competition	0.076	0.004	
**(d)**	ϕ_1	ϕ_2	ϕ_3
Birth Rate	115.278	29.121	-5.846

*This parameter includes all factors that prevent progression to the larval stage, including mortality, unfertilised eggs, etc. For simplicity it is referred to as egg mortality.
**Mortalities in low temperatures. An additional term for high temperatures is included in the model (see Fig S3b).

3 Text S3 – Population Density and Continuity

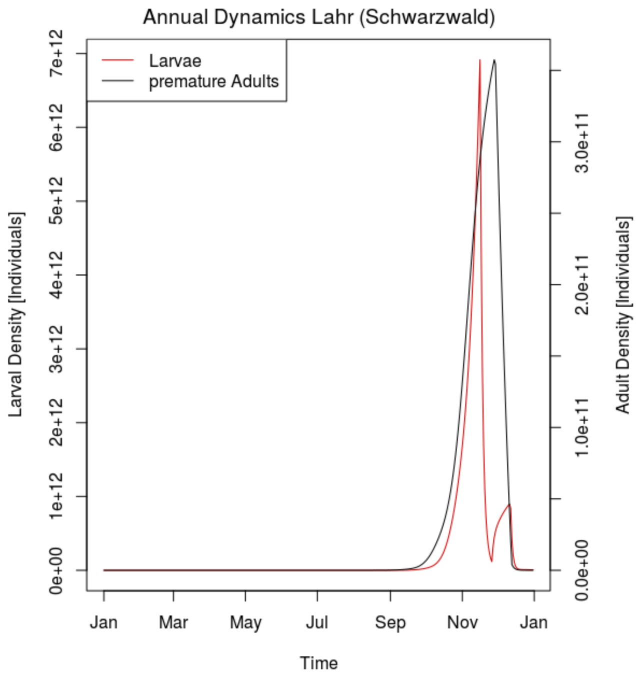

Figure S7 – Population Dynamics if no larval competition is applied.

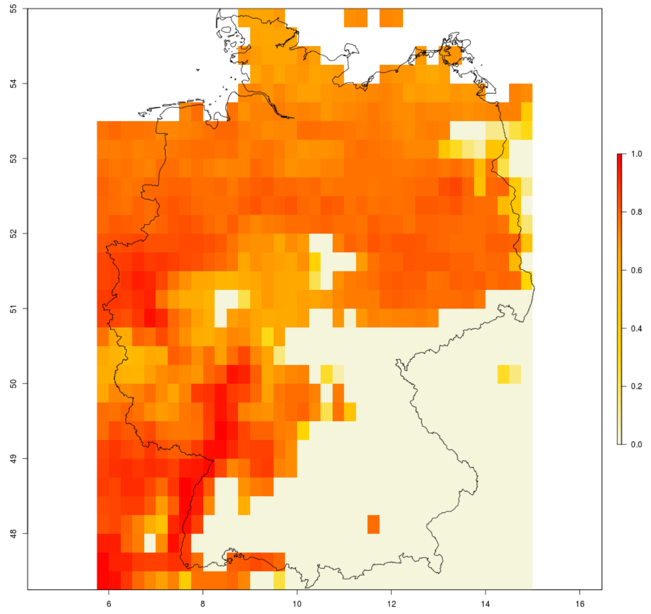

Figure S8 – Cumulative larval density without any control measure.

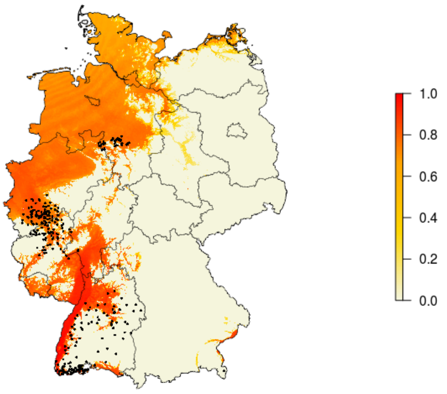

Figure S9 – Our model compared with occurrence data from Schaffner et al. (2009); Becker et al. (2013); Schneider et al. (2011); Kampen et al. (2012); Werner and Kampen (2013); Huber et al. (2014); Melaun et al. (2015); Zielke et al. (2014, 2016); Kampen et al. (2015); Bock et al. (2015)

4 Text S4 – Control Measures

Table S3 – Different scenarios in pest control measures. For every time frame (a), control of life cycle parameter (b) was performed.

**a**	**declaration**	**definition of time frame**
	constant	all year
	early spring	early year, when temperature is between 5°C and 9°C
	late spring	early year, when temperature is between 9°C and 14°C
	summer	hottest 30 days
	early fall	late year, when temperature is between 9°C and 14°C
	late fall	late year, when temperature is between 5°C and 9°C
**b**	**parameter**	**description**
	oviposition	reduced number of eggs produced per female
	ovicide	elevated mortality rates in the egg stage
	larvicide	elevated mortality rates in the larval stage
	adulticide	elevated mortality rates in the premature and adult stage

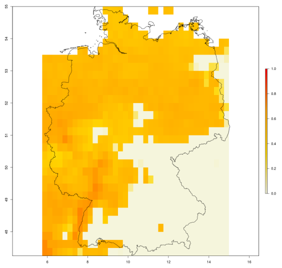

constant control (oviposition reduced by 50%)

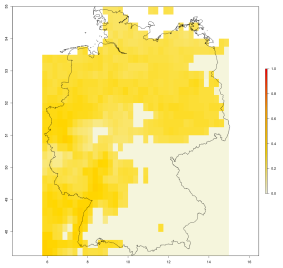

constant control (oviposition reduced by 80%)

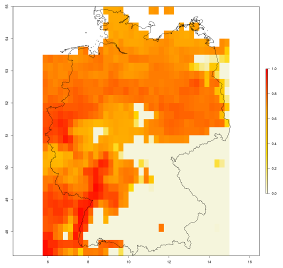

control in early spring (50%)

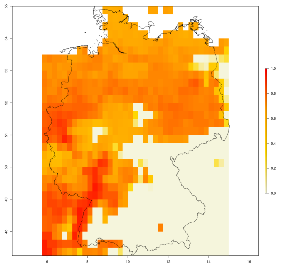

control in early spring (80%)

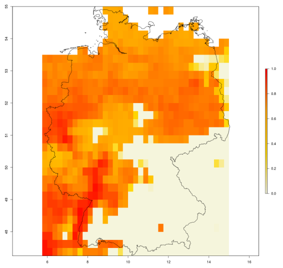

control in late spring (50%)

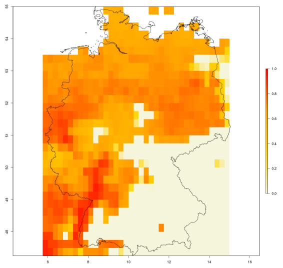

control in late spring (80%)

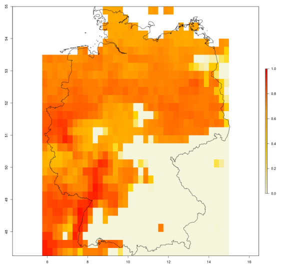

control in summer (50%)

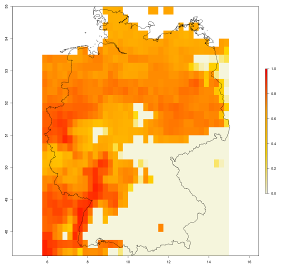

control in summer (80%)

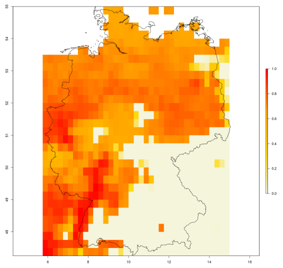

control in early fall (50%)

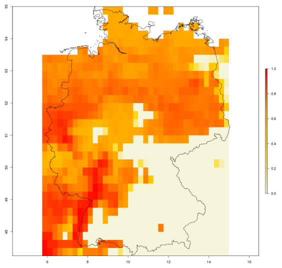

control in early fall (80%)

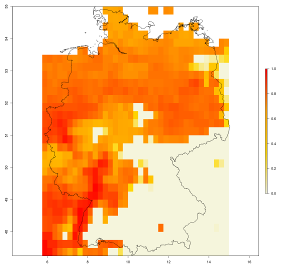

control in late fall (50%)

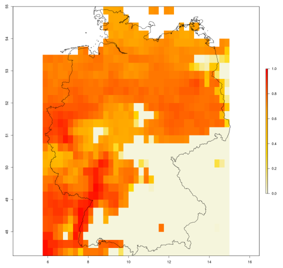

control in late fall (80%)

Figure S10 – Annual cumulative density of larvae if control measure targets oviposition. Density normalized by the highest density in scenario without any control.

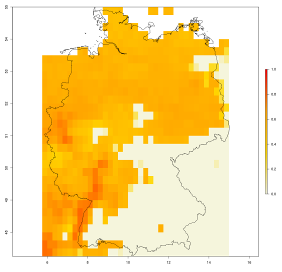

constant control (egg mortality raised by 50%)

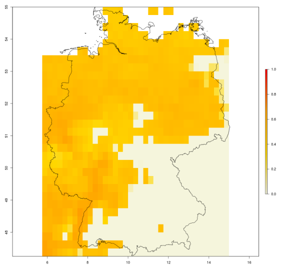

constant control (egg mortality raised by 80%)

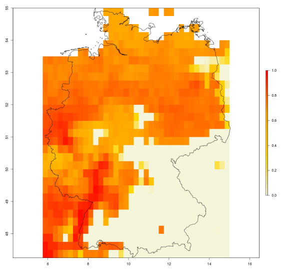

control in early spring (50%)

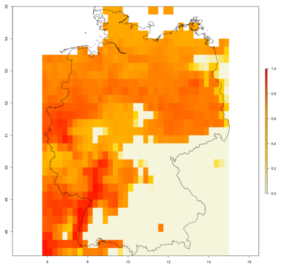

control in early spring (80%)

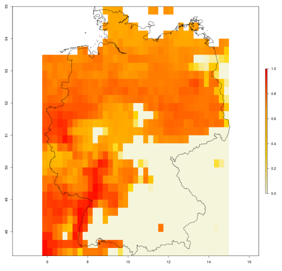

control in late spring (50%)

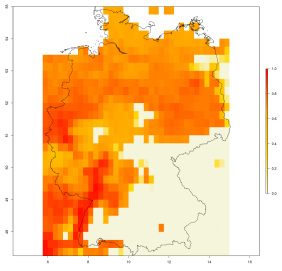

control in late spring (80%)

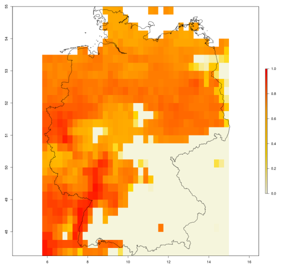

control in summer (50%)

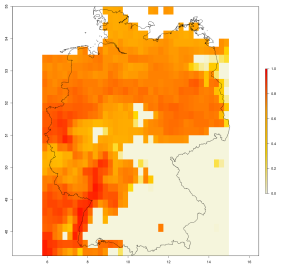

control in summer (80%)

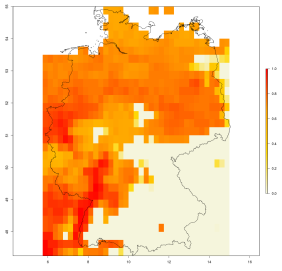

control in early fall (50%)

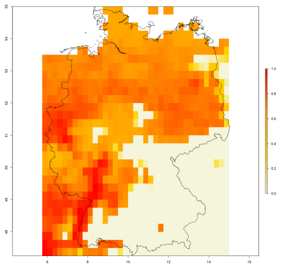

control in early fall (80%)

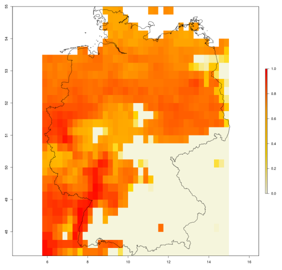

control in late fall (50%)

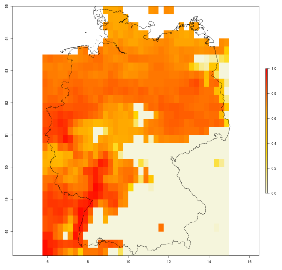

control in late fall (80%)

Figure S11 – Annual cumulative density of larvae if control measure targets egg mortality. Density normalized by the highest density in scenario without any control.

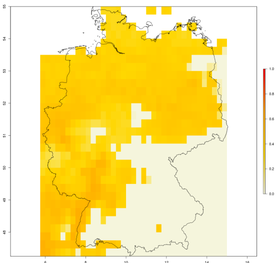

constant control (larval mortality raised by 50%)

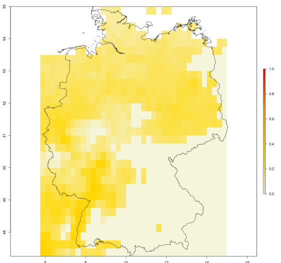

constant control (larval mortality raised by 80%)

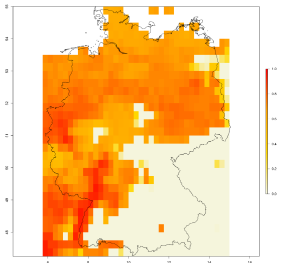

control in early spring (50%)

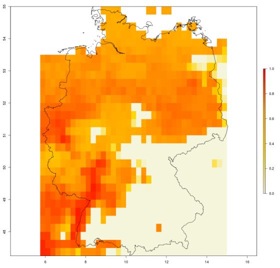

control in early spring (80%)

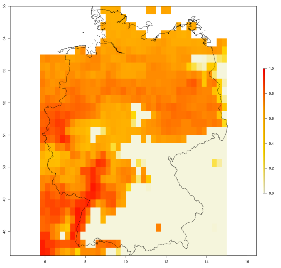

control in late spring (50%)

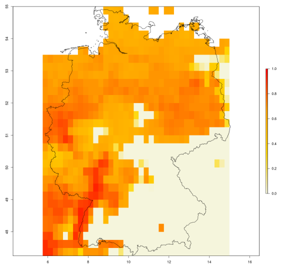

control in late spring (80%)

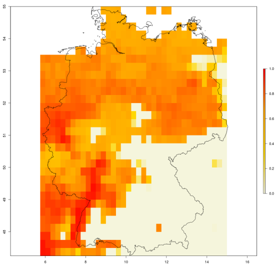

control in summer (50%)

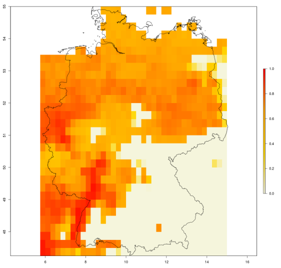

control in summer (80%)

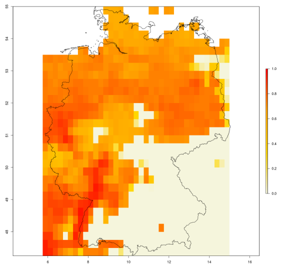

control in early fall (50%)

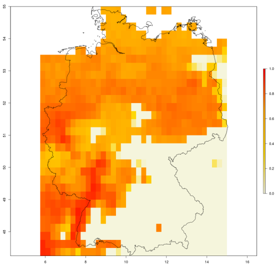

control in early fall (80%)

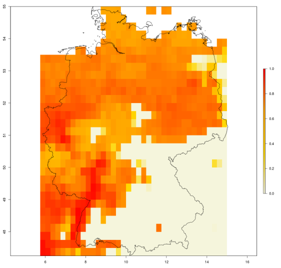

control in late fall (50%)

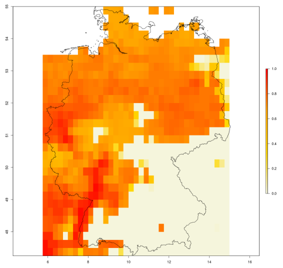

control in late fall (80%)

Figure S12 – Annual cumulative density of larvae if control measure targets larval mortality. Density normalized by the highest density in scenario without any control.

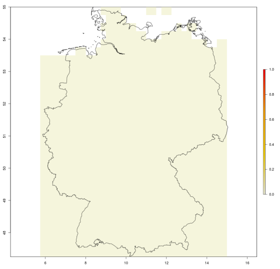

constant control (adult mortality raised by 50%)

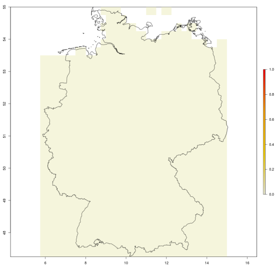

constant control (adult mortality raised by 80%)

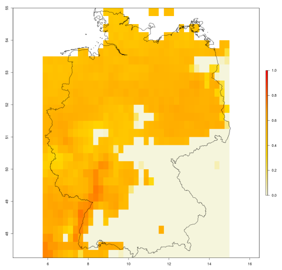

control in early spring (50%)

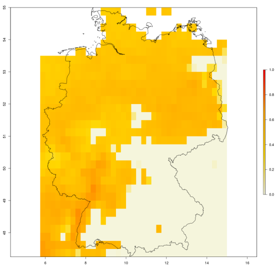

control in early spring (80%)

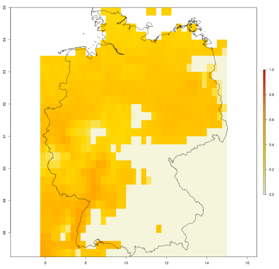

control in late spring (50%)

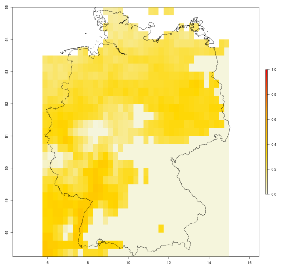

control in late spring (80%)

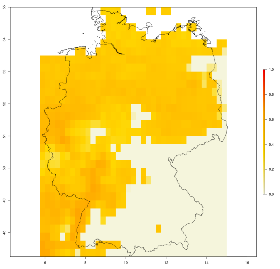

control in summer (50%)

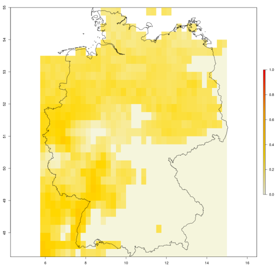

control in summer (80%)

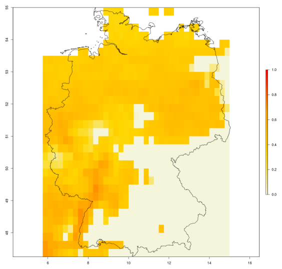

control in early fall (50%)

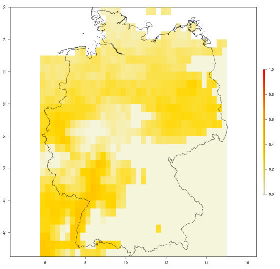

control in early fall (80%)

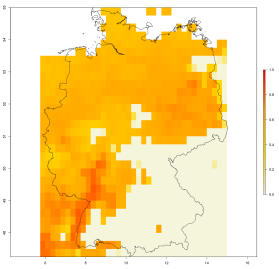

control in late fall (50%)

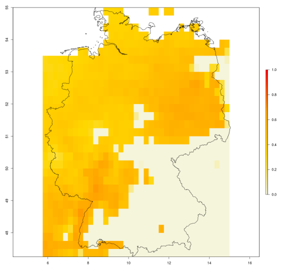

control in late fall (80%)

Figure S13 – Annual cumulative density of larvae if control measure targets adult mortality. Density normalized by the highest density in scenario without any control.

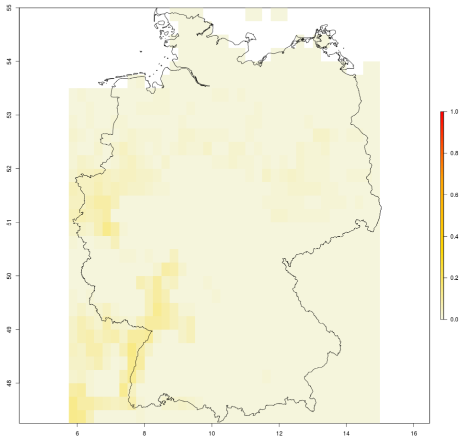

Figure S14 – Combination of constant larval control (50%) and summer adult control (50%)

Table S4 – Results of control measures. Mean values.

(a)	oviposition	egg	larva	adult
C50	0.6638	0.7278	0.4889	0
C80	0.3078	0.6034	0.232	0
ES50	0.9714	0.9702	0.9401	0.682
ES80	0.9372	0.9564	0.9232	0.6114
LS50	0.9631	0.9732	0.9274	0.5399
LS80	0.9143	0.9568	0.8941	0.2985
S50	0.957	0.9672	0.9319	0.5513
S80	0.9182	0.9533	0.9062	0.2594
EF50	0.9676	0.9684	0.9436	0.5458
EF80	0.9593	0.9614	0.9177	0.2498
LF50	0.9877	0.9875	0.9788	0.7495
LF80	0.9802	0.9839	0.9714	0.5801
(b)	mean reduction			
C50	0.3362	0.2722	0.5111	1
C80	0.6922	0.3966	0.768	1
ES50	0.0286	0.0298	0.0599	0.318
ES80	0.0628	0.0436	0.0768	0.3886
LS50	0.0369	0.0268	0.0726	0.4601
LS80	0.0857	0.0432	0.1059	0.7015
S50	0.043	0.0328	0.0681	0.4487
S80	0.0818	0.0467	0.0938	0.7406
EF50	0.0324	0.0316	0.0564	0.4542
EF80	0.0407	0.0386	0.0823	0.7502
LF50	0.0123	0.0125	0.0212	0.2505
LF80	0.0198	0.0161	0.0286	0.4199

5 Text S5 – Discussion of Winter Mortality

When temperatures are below 0 °C for more than three days, all larvae (and presumably pupae) die (Reuss et al., 2018). To ensure that our model reflects this hard border, we included special conditions for mortality during winter:
Condition 1 Our model states that larval and pupal mortality is 1, as long as temperature is below 0°C.
As can be seen in Fig. S1, the smoothed temperature function cannot always reflect freezing events. However, as the E-OBS dataset shows (Reuss et al., 2018), there is on average at least one period of three consecutive days below 0 °C. Thus, we decided to include a second condition as an 'artificial' freezing event:
Condition 2 On the three days of lowest temperature, larval and pupal mortality is 1.
Both conditions apply to every grid cell, but the requirements for Condition 1 are not always met.
Ultimately, it became clear that Condition 2 is not necessary. Its impact on the results is minimal (as can be seen in Fig. S15 and S16 densities without Condition 2 are slightly higher). However, it does not change the distribution of high risk areas, it only makes the model more complicated without actual gain. We thus would recommend to cut Condition 2 from the model, which could speed up the simulations. Additionally, adding this condition adds assumptions about freezing events, which are not necessary.

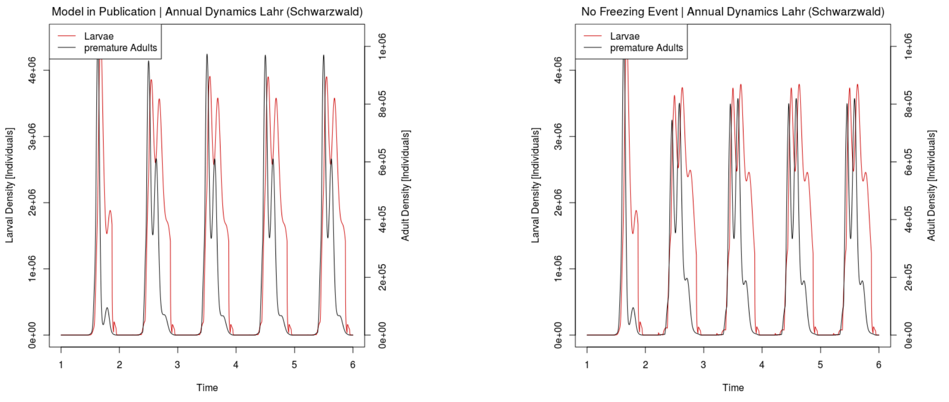

Scenario as described in main text

Scenario without artificial freezing event

Figure S 15 – Annual population density dynamics of larvae in scenarios with different winter mortality.

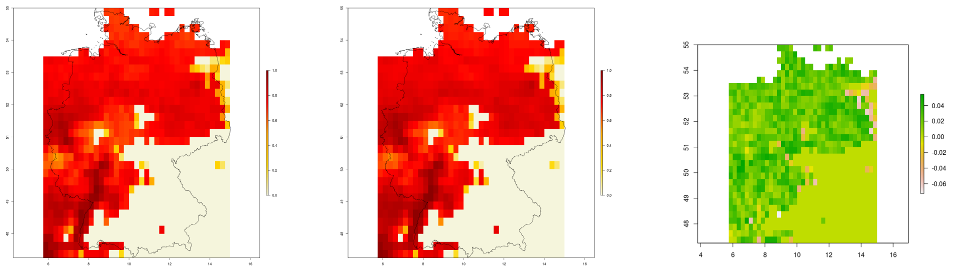

Scenario as described in main text

Scenario without artificial freezing event

Difference between scenarios

Figure S 16 – Annual cumulative density of larvae in scenarios with different winter mortality. Density normalized by the highest density in scenario without any control.

References

JS Armistead, JR Arias, N Nishimura, and LP Lounibos. Interspecific larval competition between aedes albopictus and aedes japonicus (diptera: Culicidae) in northern virginia. *Journal of medical entomology*, 45(4): 629–637, 2008.

Norbert Becker, Martin Geier, Carsten Balczun, Udo Bradersen, Katrin Huber, Ellen Kiel, Andreas Krüger, Renke Lühken, Claus Orendt, Anita Plenge-Bönig, et al. Repeated introduction of aedes albopictus into germany, july to october 2012. *Parasitology research*, 112(4):1787–1790, 2013.

Friederike Bock, Ulrich Kuch, Markus Pfenninger, and Ruth Müller. Standardized laboratory feeding of larval aedes japonicus japonicus (diptera: Culicidae). *Journal of Insect Science*, 15(1):144, 2015.

William Gurney and Roger M Nisbet. *Ecological dynamics*. Oxford University Press, 1998.

MR Haylock, N Hofstra, AMG Klein Tank, EJ Klok, PD Jones, and M New. A european daily high-resolution gridded data set of surface temperature and precipitation for 1950–2006. *Journal of Geophysical Research: Atmospheres*, 113(D20), 2008.

Robert J Hijmans, Susan E Cameron, Juan L Parra, Peter G Jones, and Andy Jarvis. Very high resolution interpolated climate surfaces for global land areas. *International journal of climatology*, 25(15):1965–1978, 2005.

Katrin Huber, Stephanie Jansen, Mayke Leggewie, Marlis Badusche, Jonas Schmidt-Chanasit, Norbert Becker, Egbert Tannich, and Stefanie C Becker. Aedes japonicus japonicus (diptera: Culicidae) from germany have vector competence for japan encephalitis virus but are refractory to infection with west nile virus. *Parasitology research*, 113(9):3195–3199, 2014.

Helge Kampen, Dorothee Zielke, and Doreen Werner. A new focus of aedes japonicus japonicus (theobald, 1901)(diptera, culicidae) distribution in western germany: rapid spread or a further introduction event? *Parasites & vectors*, 5(1):284, 2012.

Helge Kampen, Jolyon M Medlock, Alexander GC Vaux, Constantianus JM Koenraadt, Arnold JH Van Vliet, Frederic Bartumeus, Aitana Oltra, Carla A Sousa, Sébastien Chouin, and Doreen Werner. Approaches to passive mosquito surveillance in the eu. *Parasites & vectors*, 8(1):9, 2015.

Dirk Nikolaus Karger, Olaf Conrad, Jürgen Böhner, Tobias Kawohl, Holger Kreft, Rodrigo Wilber Soria-Auza, Niklaus E Zimmermann, H Peter Linder, and Michael Kessler. Climatologies at high resolution for the earth's land surface areas. *Scientific data*, 4:170122, 2017.

Helen Kettle and David Nutter. stagepop: modelling stage-structured populations in r. *Methods in Ecology and Evolution*, 6(12):1484–1490, 2015.

Christian Melaun, Antje Werblow, Sarah Cunze, Sina Zotzmann, Lisa K Koch, Heinz Mehlhorn, Dorian D Dörge, Katrin Huber, Oliver Tackenberg, and Sven Klimpel. Modeling of the putative distribution of the arbovirus vector ochlerotatus japonicus japonicus (diptera: Culicidae) in germany. *Parasitology research*, 114(3):1051–1061, 2015.

Friederike Reuss, Andreas Wieser, Aidin Niamir, Miklós Bálint, Ulrich Kuch, Markus Pfenninger, and Ruth Müller. Thermal experiments with the asian bush mosquito (aedes japonicus japonicus)(diptera: Culicidae) and implications for its distribution in germany. *Parasites & vectors*, 11(1):81, 2018.

F Schaffner, C Kaufmann, D Hegglin, and A Mathis. The invasive mosquito aedes japonicus in central europe. *Medical and veterinary entomology*, 23(4):448–451, 2009.

Katharina Schneider et al. Breeding of ochlerotatus japonicus japonicus (diptera: Culicidae) 80 km north of its known range in southern germany. *Eur Mosq Bull*, 29:129–132, 2011.

Doreen Werner and Helge Kampen. The further spread of aedes japonicus japonicus (diptera, culicidae) towards northern germany. *Parasitology research*, 112(10):3665–3668, 2013.

Dorothee E Zielke, Doreen Werner, Francis Schaffner, Helge Kampen, and Dina M Fonseca. Unexpected patterns of admixture in german populations of aedes japonicus japonicus (diptera: Culicidae) underscore the importance of human intervention. *PLoS One*, 9(7):e99093, 2014.

Dorothee E Zielke, Doreen Walther, and Helge Kampen. Newly discovered population of aedes japonicus japonicus (diptera: Culicidae) in upper bavaria, germany, and salzburg, austria, is closely related to the austrian/slovenian bush mosquito population. *Parasites & vectors*, 9(1):163, 2016.
